# The Heat Shock Protein 60 and Pap1 Participate in the *Sporothrix* *schenckii*-Host Interaction

**DOI:** 10.3390/jof7110960

**Published:** 2021-11-12

**Authors:** Laura C. García-Carnero, Roberta Salinas-Marín, Nancy E. Lozoya-Pérez, Katarzyna Wrobel, Kazimierz Wrobel, Iván Martínez-Duncker, Gustavo A. Niño-Vega, Héctor M. Mora-Montes

**Affiliations:** 1 Departamento de Biología, División de Ciencias Naturales y Exactas, Campus Guanajuato, Universidad de Guanajuato, Noria Alta s/n, col. Noria Alta, C.P., Guanajuato 36050, Gto., Mexico; laura_cgc@hotmail.com (L.C.G.-C.); nelppat@hotmail.com (N.E.L.-P.); gustavo.nino@ugto.mx (G.A.N.-V.); 2 Laboratorio de Glicobiología Humana y Diagnóstico Molecular, Centro de Investigación en Dinámica Celular, Instituto de Investigación en Ciencias Básicas y Aplicadas, Universidad Autónoma del Estado de Morelos, Cuernavaca 62209, Mor., Mexico; rsm@uaem.mx (R.S.-M.); duncker@uaem.mx (I.M.-D.); 3 Departamento de Química, División de Ciencias Naturales y Exactas, Campus Guanajuato, Universidad de Guanajuato, Noria Alta s/n, col. Noria Alta, C.P., Guanajuato 36050, Gto., Mexico; katarzyn@ugto.mx (K.W.); kazimier@ugto.mx (K.W.)

**Keywords:** sporotrichosis, cell wall, adhesin, glycoprotein, recombinant protein, virulence, moonlighting protein

## Abstract

*Sporothrixschenckii* is one of the etiological agents of sporotrichosis, a worldwide-distributed subcutaneous mycosis. Its cell wall contains a glycoconjugate composed of rhamnose, mannose, glucuronic acid, and proteins, named peptidorhamnomannan, which harbors important *Sporothrix*-specific immunogenic epitopes. Although the peptidorhamnomannan carbohydrate moiety has been extensively studied, thus far, little is known about the protein core. Here, using LC-MS/MS, we analyzed the *S.* *schenckii* peptidorhamnomannan peptide fraction and generated mass signals of 325 proteins, most of them likely to be moonlighting proteins. Among the identified proteins, chaperonin GroEL/Hsp60 and the uncharacterized protein Pap1 were selected for further analysis. Both proteins were heterologously expressed in bacteria, and they showed adhesive properties to the extracellular matrix proteins laminin, elastin, fibrinogen, and fibronectin, although Pap1 also was bound to type-I and type-II collagen. The inoculation of concentrations higher than 40 μg of these proteins, separately, increased immune effectors in the hemolymph of *Galleria* *mellonella* larvae and protected animals from an *S.* *schenckii* lethal challenge. These observations were confirmed when yeast-like cells, pre-incubated with anti-rHsp60 or anti-rPap1 antibodies were used to inoculate larvae. The animals inoculated with pretreated cells showed increased survival rates when compared to the control groups. In conclusion, we report that Hsp60 and Pap1 are part of the cell wall peptidorhamnomannan, can bind extracellular matrix components, and contribute to the *S.* *schenckii* virulence. To our knowledge, this is the first report about moonlighting protein in the *S.* *schenckii* cell wall with an important role during the pathogen–host interaction.

## 1. Introduction

Sporotrichosis is a subcutaneous mycosis with a wide range of clinical forms, spanning from localized cutaneous to disseminated infections [1,2]. The etiological agents are members of the pathogenic clade of the *Sporothrix* genus [3], and the most frequently isolated species from human and animal cases of sporotrichosis are *Sporothrix schenckii*, *Sporothrix brasiliensis*, and *Sporothrix globosa* [2]. These are thermodimorphic fungi that grow as mycelium at 25 °C, known as the saprophytic phase, and as cigar-shaped, yeast-like cells at 35–37 °C, known as the pathogenic phase [4]. These species have different virulence degrees and geographical distribution, *S. brasiliensis* being the most virulent species and restricted to Brazil and Argentina [5,6,7], followed by *S. schenckii* and *S. globosa*, cosmopolitan species but mainly found in America and Asia [2,7,8].

The fungal cell wall is a complex and essential structure that maintains cell viability, defines shape and morphology, and protects against external stresses [9]. In pathogenic species, it works as a scaffold to display virulence factors, such as hydrolases and adhesins, and is one of the first fungal structures entering in contact with host immune effectors, stimulating an antifungal immune response, but at the same time, contributing to the disguising strategies to avoid immune sensing [10,11,12,13].

The *S. schenckii* cell wall is composed of chitin in the innermost layer, followed by β-(1,3)- and β-(1,4)-glucans, and glycoproteins at the outermost layer [14,15,16,17,18]. Among the cell wall glycoproteins, a complex named peptidorhamnomannan (PRM) was identified, and it was established that the peptide core is modified with rhamnose (33.5%), mannose (57%), and galactose (1%) [14,15,19,20], which were isolated from the yeast-like cells and culture medium [19].

The first PRM analyses were performed by methylation, partial acid hydrolysis, and binding to the lectin concanavalin A (ConA), and it was indicated that 15% of mannose residues and most of the rhamnose residues are in terminal positions in the nonreducing ends of chains [19]. Further analysis showed that binding to ConA was specifically associated with mannose residues found in *O*-linked glycans [21,22], and although this reactivity was observed in the cell wall of both fungal morphologies [22], the yeast phase has a stronger affinity for the lectin when compared with hyphae [23]. This ConA reactivity was later found to be due to the presence of *O*-linked tetra- and pentasaccharides with the structures α-L-Rhap-(1-4)-α-D-GlcAp and α-L-Rhap-(1-4)-α-D-GlcAp linked to an internal core of α-D-Man-(1-2)-α-D-Man [21,24], which was confirmed by the β-elimination of *O*-linked oligosaccharides, which abolished most of the PRM reactivity to ConA and its antibody recognition [23,25]. In addition, other important structures were described to be *N*-linked glycosylated, composed of α-L-Rhap-(1-2)-α-L-Rhap-(1-3) and α-L-Rhap-(1-3) linked to an internal core of α-D-Man-(1-6) [20,26]. These PRM epitopes have not been reported in any other fungal species thus far, and therefore have been considered to be useful for the *Sporothrix* spp. serological recognition [27,28].

Rhamnose is an uncommon fungal cell wall component, and in medically relevant species, it was only reported in *Sporothrix* spp. and *Pseudallescheria boydii*; in the latter, it was also found as part of PRM [17,29]. In *S. schenckii*, it was recently shown that cell wall rhamnose content correlates with virulence [18,30], and the absence of this monosaccharide affects cell wall composition and organization and interaction with innate immune cells in a TLR4-dependent mechanism [30]. On the other hand, it was proposed that PRM is recognized by cytokine modulated Ca^2+^-dependent cell adhesion molecules [31].

Although the carbohydrate moiety of this complex is one of the best-characterized components in the *S. schenckii* cell wall, there is limited information about the proteins that compose PRM. The PRM peptide moiety is characterized by a high content in serine, threonine, alanine, and glutamic and aspartic acids, with low amounts of cysteine, methionine, and basic and aromatic amino acids [19]. However, the identity of the proteins was unknown.

Some intracellular proteins are found on the cell surface of pathogenic fungi, such as *Candida* spp. [32,33,34,35], *Histoplasma capsulatum* [36,37], *Paracoccidioides brasiliensis* [38], and *Cryptococcus neoformans* [39]. These moonlighting proteins usually have housekeeping functions inside the cell [40] but are thought to be transported to the cell wall through nonclassical secretion routes, such as vesicular transport [41] and post-translational translocation, where they can participate as virulence factors [42]. Thus, it is likely both canonical cell wall and moonlighting proteins could be part of PRM.

To get further information on the proteins that compose PRM, we analyzed this cell wall complex by mass spectrometry, and from the identified proteins, we selected two of them to assess their contribution to the *S. schenckii*–host interaction.

## 2. Materials and Methods

### 2.1. Strains and Culture Conditions

The *S. schenckii* strain ATCC MYA-4821, with a genome sequence already available [43] was used in this study. Mycelia was propagated at 28 °C in YPD, pH 4.5 (1% (*w*/*v)* yeast extract, 2% (*w*/*v*) gelatin peptone, and 3% (*w*/*v)* dextrose); whereas yeast-like cells were grown in YPD, pH 7.8, as previously reported [17]. For gene expression in bacteria, *Escherichia coli* BL21 Star (DE3) (Thermo Fisher Scientific, Waltham, MA, USA) was grown in LB broth (0.5% (*w*/*v)* yeast extract, 1% (*w*/*v)* gelatin peptone, and 0.5% (*w*/*v)* NaCl) at 37 °C. When required, media were supplemented with 2% (*w*/*v*) agar for growth on solid plates.

### 2.2. Peptidorhamnomannan Extraction and Fractioning

The extraction was performed essentially as described elsewhere [19]. Briefly, 400 mL of YPD, pH 7.8, containing 4-day-old yeast-like cells were harvested by centrifuging, washed three times with deionized water, and autoclaved at 15 psi for 30 min. After cooling, the preparation was centrifuged at 1370× *g* for 10 min and 10 °C. The supernatant was saved and precipitated with ethanol and sodium acetate 1% (*w*/*v*). The precipitate was saved, resuspended in deionized water, and dialyzed against the same solvent. This solution was precipitated again with ethanol and sodium acetate; the precipitate saved, washed with 95% (*v*/*v*) ethanol, and concentrated in a Savant SpeedVac vacuum concentrator (Thermo Fisher Scientific, Waltham, MA, USA). The sample was resuspended in 10 mL deionized water, and 5 mL Cetavlon 8% (*w*/*v*; N,N-cetyl hexadecyltrimethylammonium bromide; Sigma-Aldrich, St. Louis, MO, USA) was included, then incubated overnight at room temperature. The precipitate was then fractionated in two: an acidic fraction or fraction A, precipitated by Cetavlon directly, and fraction B, resuspended in 1% (*w*/*v*) boric acid-NaOH, pH 8.8, and left to precipitate overnight at room temperature. The recovered precipitate was centrifuged and washed with 0.5% (*w*/*v*) sodium borate, pH 8.8, and the pellet was dissolved in 50 mL of 2% (*v*/*v*) acetic acid, 1 g of sodium acetate, and 3 volumes of ethanol. The resulting precipitate was removed by centrifugation and washed once with 2% (*v*/*v*) acetic acid in ethanol and once with neat ethanol [44]. The preparation was next concentrated in a Savant SpeedVac vacuum concentrator and purified by ion-exchange chromatography on a DEAE–Sephadex column (Bio-Rad Laboratories, Hercules, CA, USA), using a linear gradient of 0–0.5 M NaCl. The fractions eluted with 0.3 M NaCl were pooled, dialyzed, lyophilized, and considered as the PRM extract [19], which was further fractionated by gel filtration on Sephadex G-100 (0.5 × 20 cm, Bio-Rad Laboratories, Hercules, CA, USA) [19], then by affinity chromatography in a ConA-Sepharose 4B column (0.5 × 20 cm, Bio-Rad), eluted with 50 mM phosphate-buffered saline (PBS), pH 7.2, then with 0.1 M methyl-α-D-mannopyranoside (Sigma-Aldrich) to detach the ConA-bound material [23]. The lectin-interacting material was saved and separated in a Bio-Gel P-2 column (1 × 100 cm, Bio-Rad). The void volume fractions were saved, lyophilized, and considered as purified PRM [23].

### 2.3. Peptidorhamnomannan Analysis by Liquid Chromatography-Mass Spectrometry

The PRM fraction was deglycosylated as follows: aliquots containing 400 µg freeze-dried PRM were resuspended in 900 µL sterile deionized water, 100 µL 3 M sodium acetate, and 25 U endoglycosidase H (New England Biolabs, Ipswich, MA, USA) and incubated overnight at 37 °C [45]. Then, pH was neutralized and samples lyophilized, then resuspended in 1 mL 100 mM NaOH, and incubated overnight at room temperature and gentle shaking [46]. The pH was neutralized with 100 mM HCl and lyophilized again. Then, 200 µg were solubilized in 100 µL of 100 mM ammonium bicarbonate buffer, pH 8.0, and reduced with 20 µL of 50 mM DTT for 1 h at room temperature [47]. Then, samples were alkylated with 20 µL of 100 mM iodoacetamide at room temperature in darkness and reduced again with 20 µL of 50 mM DTT. For hydrolysis, 20 µL of trypsin (0.25 µg µL^−1^) was used and incubation at 37 °C was carried out overnight to then stop the reaction with 20 µL of 50% (*v*/*v*) formic acid [47]. The samples were saved at −20 °C and centrifuged at 18,000× *g* for 20 min at 4 °C before being analyzed by capillary liquid chromatography-electrospray ionization-quadrupole time-of-flight mass spectrometry (capLC-ESI-QTOF-MS), using an UltiMate 3000 RSLC (Thermo Fisher Scientific) coupled to a maXis impact mass spectrometer (Bruker Daltonics, Billerica, MA, USA), as previously described [47]. Briefly, aliquots of 5 µL PRM were loaded on a C18 capillary trap (5 μm, 100 Å, 300 μm i.d. × 5 mm, Agilent Technologies, Santa Clara, CA, USA) using 0.1% (*v*/*v*) formic acid and 2% (*v*/*v*) acetonitrile at a flux rate of 15 μL min^−1^ for 2 min. Then, the flux was changed to the reversed phase capillary column (Halo C18, 150 × 0.3 mm, 2.7 μm), and the separation was carried out at 40 °C and a flux rate of 3 μL min^−1^ using two mobile phases (A-aquos 0.1% (*v*/*v*) formic acid; B-0.1%(*v*/*v*) formic acid in acetonitrile) with a linear gradient of 2 to 80% (*v*/*v*) B for 20 min. The column was connected to an electrospray ionization source via 50 cm capillary (i.d. 50 μm), introducing the lock-mass standard *m*/*z* 1221.9907 in the ion source. ESI was operated in a positive mode with ion spray voltage 4500 V, endplate offset 500 V, dry gas 4 L min^−1^, drying temperature 180 °C, and nebulizing gas pressure 0.4 bar. MS data were obtained with an acquisition rate of 2 Hz within the 300–2200 *m/z* range. For auto-MS/MS mode, filtration for amino acids was applied with an acquisition rate of 2 Hz and 20 Hz for low (<25,000) and high (>25,000) ion counts, respectively. Line spectra calculated as a sum of intensities were always registered. For protein identification and for label-free quantification, LC-MS and MS/MS data were analyzed in MaxQuant (version 1.5.5.1) [48] using the *S. schenckii* taxonomy database from UniProtKB (https://www.uniprot.org/proteomes/UP000033710; accessed on 2 March 2018). The following parameters were applied: trypsin as specific digestion enzyme, methionine oxidation and acetylation of N-terminals as variable modification, and carbamidomethylation of cysteines as fix modification. Minimum and maximum peptide charge states were set at +2 and +5, respectively. The initial precursor mass tolerance of 6 ppm and fragment mass tolerance of 0.01 Da were applied. The maximum false discovery rate for peptides and proteins was 1% based on comparison to a reverse database; seven amino acids was the minimum length of peptide. A time window of 0.7 min with an alignment time window of 15 min were selected for time matching between LC-MS/MS runs. Proteins that matched typical contaminants or those included in the reverse database were excluded.

### 2.4. Peptidorhamnomannan Electrophoresis and Lectin Blot

Aliquots of 75 µg PRM were subjected to denaturing sodium dodecyl sulfate-polyacrylamide gel electrophoresis (SDS-PAGE) using 12% (*w*/*v*) polyacrylamide gels and separated at 100 V for 90 min. The separated material was either used for conventional staining with Coomassie Brilliant Blue G-250 Dye (Thermo Fisher Scientific) or transferred to a nitrocellulose membrane for blotting. The membrane was blocked with 5% (*w*/*v*) polyvinylpyrrolidone (Sigma-Aldrich) in TBS buffer (50 mM Tris-HCl, pH 7.6, 150 mM NaCl) for 60 min, then was incubated for 2 h at room temperature with 1 mg mL^−1^ ConA-fluorescein (FITC; Sigma-Aldrich) in TBS added with 0.1% (*v*/*v*) Tween 20 (Sigma-Aldrich). Then, the membrane was washed six times with TBS-Tween and inspected in a ChemiDoc MP (Bio-Rad), using a wavelength of 520 nm.

### 2.5. Bioinformatic Analysis

Putative sites of *N*-linked and *O*-linked glycosylation were predicted using the GPP prediction server (https://comp.chem.nottingham.ac.uk/cgi-bin/glyco/bin/getparams.cgi; accessed on 5 October 2018); signal peptide prediction was performed with SignalP 4.1 server [49]; and the disordered domains were predicted using the IUPred2A server (https://iupred2a.elte.hu; accessed on 5 October 2018) [50].

### 2.6. RNA Isolation and Gene-Expression Analysis

Total RNA was isolated as previously reported from either mycelia or yeast-like cells [51,52]. Alternatively, HeLa cells (ATCC) were grown in Eagle’s Minimum Essential Medium (EMEM, Sigma-Aldrich) at 37 °C and 5% CO_2_ (*v*/*v*) until monolayer generation. Then, cells were treated with 0.25% (*w*/*v*) trypsin and 0.53 mM EDTA (Sigma-Aldrich) until monolayer detachment, cells were washed twice with phosphate-buffered saline (PBS), concentration was adjusted to 5 × 10^6^ cells mL^−1^ with EMEM, and 200 µL were placed in flat-bottom 96-well microplates. Then aliquots of 200 µL of yeast-like cells at a cell concentration of 2 × 10^6^ cells mL^−1^ in EMEM were added and incubated for 24 h at 37 °C and 5% (*v*/*v*) CO_2_. Cells were pelleted by low-speed centrifugation and used for RNA extraction. Total cDNA was synthesized and purified by adsorption chromatography following the methodology reported elsewhere [53], quantified in a NanoDrop 2000 (Thermo Fisher Scientific), and amplified with the SYBR Green Master Mix (Thermo Fisher Scientific) in a StepOnePlus thermocycler (Thermo Fisher Scientific). Relative gene expression was calculated with the StepOne software V 2.2 (Thermo Fisher Scientific) using the 2^−∆∆Ct^ method [54]. For data normalization, the encoding gene for the ribosomal protein L6 was amplified as endogenous control [51] and mycelia as the reference condition when cells were grown in YPD. When interacting with HeLa cells, cells growing in EMEM were used as the reference condition. The following primer pairs were used in the RT-qPCR reactions: 5′-ATTGCGACATCAGAGAAGG-3′ and 5′-TCGACCTTCTTGATGTTGG-3′ for the endogenous control (amplification efficiency 98.1 ± 0.2%); 5′-GTCGTCGAGTTCCTCCAGAA-3′ and 5′-CTTCTTCTCGGACAGCAGGA-3′ for *HSP60* (amplification efficiency 97.4 ± 0.2%); 5′-GATGAGTTCGGCACCATTGG-3′ and 5′-CACAGATGGCGGCACTTG-3′ for *PAP1* (amplification efficiency 98.4 ± 0.1%).

### 2.7. Heterologous Expression of Sporothrix Schenckii HSP60 and PAP1 in Escherichia coli

Upon total RNA isolation and cDNA synthesis, as described in the previous section, the open reading frames from *HSP60* and *PAP1* were amplified by PCR using the primer pairs 5′-GAATTCATGCAGCGTGCCATGACCTC-3′ and 5′-TCTAGATTACATCATGCCGCCCATGCC-3′ for *HSP60*; and 5′-GAATTCATGTCTTCCATCGTCAACAAGAT-3′ and 5′-TCTAGATTAAATTCCCCGGAACGACTC for *PAP1* (underlined bases indicate EcoRI and XbaI sites, added to the forward and reverse primer sequences, respectively, for direct cloning). The 1770 bp and 918 bp amplicons, corresponding to the open reading frame of *HSP60* and *PAP1*, respectively, were cloned into pJET1.2/blunt (Thermo Fisher Scientific), then subcloned into the EcoRI and XbaI sites of pCold I (Takara Bio Inc, Kusatsu, Shiga, Japan), generating pCold-*HSP60* and pCold-*PAP1*, respectively. For recombinant gene expression, the constructions were used to transform *E. coli* BL21 (DE3; Thermo Fisher Scientific), and cells were grown in LB broth with 100 µg mL^−1^ ampicillin (Sigma-Aldrich) for 20 h at 37 °C and orbital shaking (200 rpm). Then, 150 mL of fresh broth contained in a 2 L Erlenmeyer flask was inoculated with 1.5 mL of the overnight culture and incubated at 37 °C until reaching an O.D. 600 nm = 0.4, then 0.1 mM isopropyl-β-D-1-thiogalactopyranoside was added and further incubated for 20 h at 15 °C. Cells were harvested by centrifuging for 20 min at 1500× *g* and 4 °C and kept at −20 °C until use.

### 2.8. Recombinant Protein Purification

Induced bacteria were washed three times with deionized water, resuspended in 5 mL of buffer A (100 mM NaH_2_PO_4_, 10 mM Tris-HCl, 8 M urea, pH 8.0), and subjected to 10 cycles of freezing in liquid nitrogen, resting at 25 °C between cycles. The cell homogenate was centrifuged at 9485× *g* for 10 min, then the supernatant was saved and loaded onto a 10 mL column containing 2 mL of TALON Metal Affinity Resin (Jena Bioscience, Jena, Germany). After a 60 min interaction at room temperature, the column was washed with buffer A at pH 8.0, 7.4, and 7.0 (three-column volumes for each pH solution), and the proteins of interest were eluted with 5 volumes of buffer A at pH 6.5 and 5 volumes of pH at 5.4. Then proteins were loaded onto a 12% (*w*/*v*) polyacrylamide gel and electrophoretically separated under denaturing conditions at 100 V for 90 min. Then, gels were washed three times with deionized water and stained with 0.25 M KCl and 1 mM dithiothreitol to visualize the protein bands and to slice out those of interest [55]. Proteins were passively diffused from polyacrylamide slices by suspending the gel pieces in PBS and incubating at 25 °C overnight with gentle shaking (120 rpm) [56]. Finally, proteins were concentrated and desalted in an Amicon Ultra centrifugal filter with Ultracel-3K (Sigma-Aldrich).

To assess purity, proteins were separated by SDS-PAGE in 12% gels, silver-stained as reported [57], and densitometrically analyzed in a ChemiDoc™MP (Bio-Rad) system. Protein quantification was performed with the Pierce BCA Protein Assay Kit (Thermo Fisher Scientific).

### 2.9. Generation of Polyclonal Antibodies against Recombinant Hsp60 and Pap1

Antibodies were generated in New Zealand rabbits as previously described [58]. The antigenic preparations contained 150 µg of either the purified rHsp60 or rPap1 and the complete Freund’s adjuvant and were intramuscularly injected. After 2 weeks, one booster dose was injected, and the procedure was repeated until four booster doses were completed. Two weeks after the last booster, animals were bled, the sera were collected, and the globulin fraction was precipitated with 76% (*w*/*v*) ammonium sulfate. Aliquots of 10 mL blood were withdrawn before the immunization protocol, and the sera were collected and kept at −20 °C until used. ELISA was used to title the antibody production, using the 96-well plates coated with 3 µg of the specific recombinant protein. The title of the antibodies generated was 1:6400 and 1:4200 for the anti-rHsp60 and rPap1 antibodies, respectively.

### 2.10. Analysis of Cell Adhesion to Extracellular Matrix Components

Two ELISA-based approaches were used to assess *S. schenckii* adhesion to extracellular matrix (ECM) components, as previously reported [59]. Nunc MaxiSorp™ flat-bottom 96-well microplates were coated with 1 µg of human laminin, human elastin, human fibrinogen, recombinant human fibronectin, recombinant human thrombospondin-1, human type-I collagen, or bovine type-II collagen (all from Sigma-Aldrich). The proteins were suspended in 0.05% (*w*/*v*) PBS Tween 20, and plates were incubated for 3 h at room temperature. Then, plates were blocked overnight with 1% (*w*/*v*) bovine serum albumin (BSA) in PBS at 4 °C. Finally, plates were washed three times with 0.05% (*w*/*v*) PBS Tween 20 and were immediately used in adhesion experiments.

Aliquots of 100 µL containing 5 × 10^6^ yeast-like cells were added to each well, and plates were incubated for 60 min at 37 °C. Plates were washed three times with 0.05% (*w*/*v*) PBS Tween 20, 100 µL of polyclonal anti-rGp70 [56] at a working dilution 1:3000 was added, and plates were incubated 2 h at room temperature. Then, plates were washed again, and 100 µL of goat anti-rabbit IgG-peroxidase antibody (1:5000; Sigma-Aldrich) was added and further incubated for 2 h at room temperature. Finally, the presence of peroxidase was revealed by incubating at room temperature for 20 min with 0.1 mg mL^−1^ 2,2′-Azino-bis(3-ethylbenzothiazoline-6-sulfonic acid) diammonium salt and 0.006% (*v*/*v*) hydrogen peroxide and stopped by adding 2 N sulfuric acid. The absorbance at 450 nm was read in a Varioskan LUX Multimode Microplate Reader (Thermo Fisher Scientific). When required, fungal cells were incubated with either polyclonal antibodies against rHsp60 or rPap1 at a working dilution of 1:3000 for 60 min at 37 °C. Then, cells were washed three times with PBS and used in the adhesion assays.

Alternatively, bacteria expressing the recombinant proteins were washed three times with PBS, resuspended in 5 mL of the same buffer added with 50 mg mL^−1^ lysozyme (Sigma-Aldrich), and incubated for 60 min at 37 °C. Then, 1% (*w*/*v*) SDS was added, and cells were disrupted by incubating for 30 min at 37 °C and shaking for 20 min in a vortex. The cell homogenate was centrifuged at 21,000× *g* at 4 °C, then 5 µg protein from the supernatant were added to wells coated with the ECM proteins, incubated 60 min at 37 °C, and washed three times with 0.05% (*w*/*v*) PBS Tween 20. Polyclonal antibodies against rHsp60 or rPap1 at a working dilution of 1:3000 were added and incubated 2 h at room temperature. The secondary goat anti-rabbit IgG-peroxidase antibody and detection of peroxidase activity were performed as above described.

In both formats, wells coated only with the blocking agent (BSA) were used as control, and the readings from these wells were subtracted from the values obtained from wells coated with ECM proteins.

### 2.11. Infection Assays in Galleria mellonella

The role of both Hsp60 and Pap1 in fungal virulence and the ability of recombinant proteins to prevent sporotrichosis was assessed in an experimental model of invasive sporotrichosis in the alternative host *G. mellonella* [56]. The use of animals in this study was approved by the Ethics Committee of Universidad de Guanajuato (permission CIBIUG-P12-2018).

Larvae were obtained from an in-house colony [60] and fed ad libitum with an already described diet based on corn bran and bee honey [7], and only those without evident body melanization and size between 1.2 and 1.5 cm were included in the study [7,60]. Fungal cells, in a concentration of 1 × 10^5^ yeast-like cells 10 µL^−1^, were injected with a Hamilton syringe equipped with a 26-gauge needle in the last left pro-leg, which was previously sanitized with 70% (*v*/*v*) ethanol, and animals were housed in Petri dishes and kept at 37 °C for 2 weeks [60]. The animal survival was monitored daily, and animal dehydration was avoided by adding chopped apple to the animal housing [7], while silk was removed daily to delay the transition to pupa. Animal death was defined as lack of irritability and extensive body melanization as previously defined [7,56,60]. Each animal group contained 30 larvae and were injected with 10 µL of PBS containing 10 µg, 20 µg, 40 µg, 80 µg, or 160 µg of recombinant protein. Larvae were incubated 5 days at 37 °C, then challenged with 1 × 10^5^ yeast-like cells, and monitored daily for 2 weeks. As controls, one animal group was injected only with PBS or only with the recombinant proteins.

When indicated, 1 × 10^5^ yeast-like cells were pre-incubated with a working dilution of 1:2000 of either polyclonal antibodies against rHsp60 or rPap1 for 60 min at 37 °C; cells were washed twice with PBS and used for animal inoculation. As controls, fungal cells were incubated with a working dilution of 1:2000 of the preimmune sera before inoculation to larvae. Moreover, animal groups were inoculated with 10 µL of the 1:2000 dilution of preimmune sera or the polyclonal antibodies against rHsp60 or rPap1.

### 2.12. Analysis of the Galleria mellonella–Sporothrix schenckii Interaction

The colony-forming units (CFUs) from hemolymph were calculated as previously reported [7], decapitating live and dead animals with a serial dilution of hemolymph incubated on YPD plates, pH 4.5, at 28 °C for 72 h.

The following insect parameters were analyzed in animal groups containing 10 animals 24 h post inoculation, as described [7]. The recovered hemolymph from inoculated and control animals was anticoagulated with 150 µL of 93 mM NaCl, 100 mM glucose, 30 mM trisodium citrate, 26 mM citric acid, 10 mM Na_2_EDTA, and 0.1 mM phenylthiourea, pH 4.6 [56], and hemocyte concentration was calculated in a hemocytometer as described [61]. For phenoloxidase quantification, protein concentration was calculated in the cell-free hemolymph, using the Pierce BCA Protein Assay Kit (Thermo Fisher Scientific), and enzyme reactions were performed in a final volume of 200 µL and contained 100 µg protein and 20 mM 3,4-dihydroxyDL-phenylalanine (Sigma-Aldrich). Reactions were placed in 96-well microplates, and the 490 nm absorbance was read in a Multiskan™ FC Microplate Photometer (Thermo Fisher Scientific). After 30 min incubation at 37 °C, absorbance was measured again and enzyme activity was defined as the Δ_490nm_ per minute per µg protein [62]. Melanin production was quantified by measuring the absorbance at 405 nm of hemolymph, as reported [63], while the released lactate dehydrogenase (LDH) activity in the cell-free hemolymph was quantified with the Pierce LDH Cytotoxicity Assay Kit (Thermo Fisher Scientific), as described [64]. For cell normalization, the LDH activity of homogenized hemocytes was quantified and referred to here as 100% cytotoxicity. In all measurements, samples from noninfected larvae or inoculated with PBS were used as controls.

### 2.13. Statistical Analysis

The GraphPad Prism 6 software was used for statistical analyses. The in vivo assays included a total of 30 larvae per group; data were plotted in Kaplan–Meier survival curves and analyzed using the log-rank test. Other results are reported as the media ± standard deviation from three independent experiments performed by duplicate and were analyzed with the Mann–Whitney U test. The statistical significance was set at *p* < 0.05 in all cases.

## 3. Results

### 3.1. Polypeptide Composition of Peptidorhamnomannan

**Figure 1:**
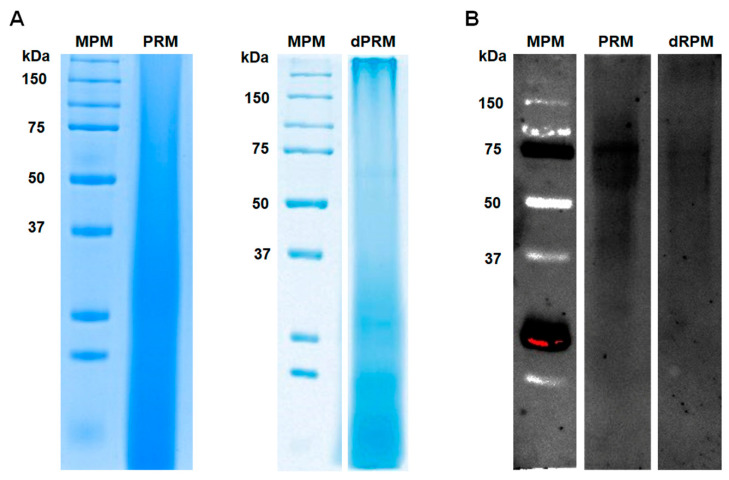
Peptidorhamnomannan analysis by denaturing electrophoresis and lectin blotting. Protein separation was carried out in 12% (*w*/*v*) polyacrylamide gels under denaturing conditions, and the separated material was either used for conventional protein staining with Commassie Blue (**A**) or was subjected to lectin blotting (**B**) using fluorenscein-conjugated concanavalin A. The membranes were inspected using a transilluminator with a wavelength set at 520 nm. MPM, molecular protein marker; PRM, peptidorhamnomannan. dPRM peptidorhamnomannan was treated with endoglycosidase H and β-elimination before electrophoresis.

Although the PRM carbohydrate composition was studied and the structure of the main glycans was already solved [19,20,21,23,24], little is known about the peptide backbone of this cell wall complex. PRM was extracted from the yeast-like cells wall, following the conventional methodology described in Material and Methods section. When this fraction was analyzed by denaturing SDS-PAGE, no defined protein bands were identified (Figure 1A), most likely due to the high degree of protein glycosylation. When the PRM was deglycosylated by treatment with β-elimination and endoglycosidase H, most of the dispersed material found in the PRM electrophoretic mobility disappeared and concentrated to the bottom of the lane and two faint protein bands of 65 kDa and 54 kDa were observed (Figure 1A). To confirm the PRM glycosylation, the sample was analyzed by lectin blot, using ConA-FITC, and under these conditions, protein bands with molecular weights in the rank of 90–55 kDa were identified, and a 75 kDa protein band being the most abundant in the PRM preparation (Figure 1B). When the lectin blot was repeated with the deglycosylated material, most of the signals observed in the glycosylated samples were lost, and the labeling of the 75 kDa protein was weaker in the deglycosylated PRM (Figure 1B).

The deglycosylated PRM was subjected to capLC-ESI-QTOF/MS and MS/MS, yielding 325 polypeptides (Supplementary Material, Table S1), and from these, 26 main proteins were identified with Andromeda scores above 100 and with *q* values equal to zero [48] (Table 1). From this table, we decided to work with the chaperonin GroEL-like protein, also known as heat shock protein 60 (Hsp60), because it was reported to be involved in the pathogen–host interaction in other fungal species and we obtained the highest sequence coverage (48.9%) and Andromeda score (323) for this protein. The protein was isolated from both prokaryotic and eukaryotic cells, and in the latter, the canonical function is performed in the mitochondria, assisting the folding of proteins with regions of exposed hydrophobic β-sheets and molecular weight lower than 60 kDa [65]. However, it was also reported as a moonlighting protein found in the cell wall of *H. capsulatum* and *P. brasiliensis*. Regarding the former, it was reported as a highly immunogenic glycoprotein that participates in adhesion to the host, and vaccination with a recombinant version protects mice from the deep-seated histoplasmosis [36,37]. Similarly, vaccination with Hsp60 protected laboratory animals from the pulmonary infection caused by *P. brasiliensis*, suggesting this protein may also be found on the cell surface of this fungal species [66]. The *S. schenckii* Hsp60 showed 99%, 96%, 96%, 96%, 95%, 91%, and 90% similarity to *S. brasiliensis*, *S. globosa*, *Ophiostoma piceae*, *Grosmannia clavigera*, *Sporothrix insectorum, H. capsulatum,* and *P. brasiliensis* Hsp60, respectively. Moreover, it was predicted to contain 38 and 5 putative sites of *O*-linked and *N*-linked glycosylation, respectively, and no signal peptide was predicted.

It was interesting to observe that six out of the 26 proteins identified in the PRM have an unknown obvious function, and we decided to work with the protein encoded by SPSK_00848, following the rationale that our results indicated a slightly higher sequence coverage percentage with this protein than that with the protein encoded by SPSK_05930. Additionally, when compared with the protein encoded by SPSK_04236, the protein encoded by SPSK_00848 (named here as Petidorhamnomannan-associated protein 1, Pap1) contains 26 and five putative *O*-linked and *N*-linked glycosylation sites, values higher than those predicted for the SPSK_04236 product (22 and two putative *O*-linked and *N*-linked glycosylation sites, respectively). *S. schenckii* Pap1 showed 79% similarity to *S. brasiliensis* Pap1, but no obvious putative ortholog could be identified in *S. globosa* and *Sporothrix insectorum*, species whose genome sequence were deposited in the GenBank of NCBI (https://www.ncbi.nlm.nih.gov/genbank/; accessed on 5 October 2018). However, putative homologs were found in *O. piceae* and *G. clavigera* (49% and 47% similarity, respectively); in the former, the polypeptide was predicted to be a cell-surface protein (GenBank accession code EPE10640). Similar to Hsp60, no signal peptide was predicted for *S. schenckii* Pap1.

The *S. schenckii* transcriptome in both filament and yeast-like cells was recently reported [67], and both *HSP60* and *PAP1* were found upregulated in yeast-like cells (log2FC values of 1.34 and 4.70, respectively) [67]. To confirm these observations, we analyzed the expression of both genes by RT-qPCR using the mycelial growth as a reference condition and the expression of the ribosomal protein L6 as a reference gene [51]. YPD-grown yeast cells showed 1.98 ± 0.15 and 5.1 ± 0.44 folds of increased expression of *HSP60* and *PAP1,* respectively, confirming the previous observation by RNA sequencing [67]. Moreover, when yeast-like cells were incubated with the HeLa cell line, under incubation conditions closer to those found within the human host, gene expression increased 2.96 ± 0.38 and 7.23 ± 0.31 folds for *HSP60* and *PAP1,* respectively, when compared to cells growing only in EMEM medium. For both genes, the expression level was statistically significant in yeast-like cells interacting with HeLa cells, when compared to YPD-grown yeast cells (*p* < 0.05). Therefore, both genes are upregulated in yeast-like cells and during interaction with the host.

### 3.2. Production of Recombinant Hsp60 and Pap1

**Figure 2:**
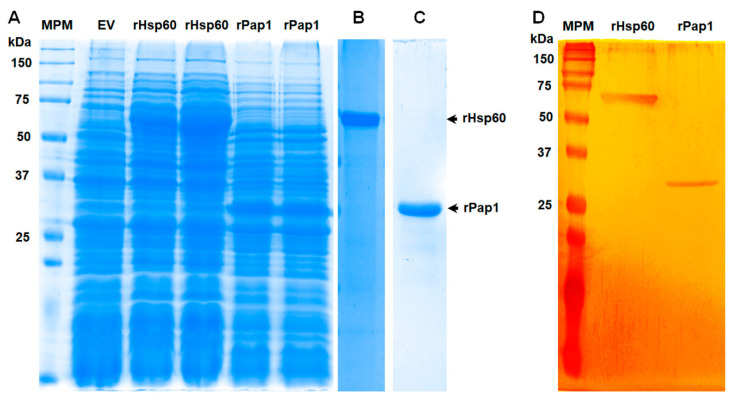
Expression of recombinant Hsp60 and Pap1 expressed in *Escherichia coli*. In (**A**), bacteria were transformed with pCold-*HSP60* (rHsp60 lanes), pCold-*PAP1* (rPap1 lanes), or the empty pCold I vector (EV) and grown under inducing condition (0.1 M isopropyl-β-D-1-thiogalactopyranoside and 20 h at 15 °C). Protein samples were prepared from cultured cells and separated by denaturing SDS-PAGE in 12% (*w*/*v*) gels. Two independent clones for production of each recombinant protein were analyzed. The recombinant proteins were then subjected to purification as described in the main text and were either Commassie Blue-stained ((**B**,**C**) correspond to purified rHsp60 and rPap1, respectively) or silver-stained (**D**). MPM, molecular protein marker.

To assess the role of both Hsp60 and Pap1 during the *S. schenckii*–host interaction, we generated recombinant proteins in a prokaryotic system and used these polypeptides for the production of polyclonal antibodies against them. The open reading frames of the genes under study were amplified from *S. schenckii* cDNA and cloned in the expression vector pCold I, generating pCold-*HSP60* and pCold-*PAP1*, respectively. Gene expression was induced in *E. coli* cells with isopropyl-β-D-1-thiogalactopyranoside and incubation at 15 °C. Several conditions for gene induction were tested in pilot experiments (not shown), and the best conditions were found to be 0.1 mM of the inductor and 20 h of incubation. Under these inducing conditions, two differentially expressed protein bands were observed in the cell homogenates, one of 65 kDa, corresponding to recombinant (r) Hsp60, and one of 32 kDa, corresponding to rPap1 (Figure 2A). Since the expression vector contained sequences for molecular tags that are part of the recombinant proteins, such as 6xHis [56], it is likely that these extra sequences were responsible for the discrepancy between the experimental and predicted molecular weights of 62 kDa and 29 kDa for Hsp60 and Pap1, respectively. As a control, cells transformed with the empty pCold I vector did not show the differentially expressed protein bands (Figure 2A), suggesting that those protein bands of 65 and 32 kDa were the recombinant rHp60 and rPap1, respectively. Since both proteins contain the 6xHis tag at the N-terminal end, protein purification was performed by affinity chromatography in a nickel-charged resin, followed by purification from SDS-PAGE gels, as reported for other proteins [56]. Following this strategy, one single protein band was observed in the purified fractions, with the corresponding 65 and 32 kDa protein bands for rHsp60 and rPap1, respectively (Figure 2B,C). The silver-stained gel lanes (Figure 2D) analyzed by densitometry indicated that more than 98.5% of protein present in the preparations corresponded to the recombinant proteins. Following this procedure, we obtained a yield of 68.0 ± 1.3 µg and 37.0 ± 1.8. µg pure rHsp60 and rPap1 per mL of induced culture media, respectively. The recombinant proteins were used to generate polyclonal antibodies, which were used in the experiments described in the following sections.

### 3.3. Hsp60 and Pap1 Participate in the Sporothrix schenckii Adhesion to Extracellular Matrix Proteins

**Figure 3:**
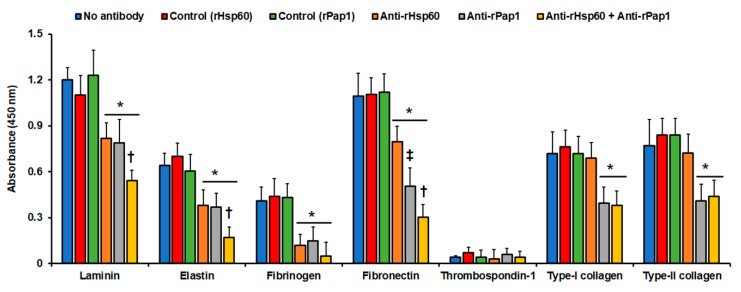
Adhesion of *Sporothrix schenckii* yeast-like cells to immobilized extracellular matrix proteins. The extracellular matrix proteins were immobilized in the 96-well plates and used in ELISA-based analyses. Yeast-like cells were added to the plates, nonadherent cells were washed, and cells bound to extracellular matrix proteins were detected with anti-rGp70 antibodies and peroxidase-conjugated anti-rabbit IgG. No antibody, cells were pre-incubated with PBS; Control (rHsp60) and Control (rPap1), cells were pre-incubated with pre-immune sera; anti-rHsp60, cells were pre-incubated with polyclonal anti-rHsp60 antibodies; and anti-rPap1, cells were pre-incubated with polyclonal anti-rPap1. Results are means ± SD of three independent experiments performed by duplicate. * *p* < 0.05 when compared to cells pre-incubated with PBS. ^†^ *p* < 0.05 when compared to cells pre-incubated with anti-rHsp60 or rPap1. ^‡^ *p* < 0.05 when compared to cells pre-incubated with anti-rHsp60.

Recombinant proteins and polyclonal anti-rHsp60 and anti-rPap1 antibodies were used in ELISA-based experiments to assess binding to immobilized laminin, fibrinogen, fibronectin, and type-II collagen. We selected these proteins because it was previously demonstrated that *S. schenckii* yeast-like cells can adhere to them [59]. As a negative control, we included thrombospondin-1, an ECM component that is not bound by these cells [59], and elastin and type-I collagen, two ECM components previously uncharacterized in terms of the *S. schenckii* ability to adhere to them. The *S. schenckii* yeast-like cells showed adhesion to all the ECM components tested with exception of thrombospondin-1, as reported [59] (Figure 3). Cells showed the highest adhesion to laminin and fibronectin, followed by type-I and type-II collagen, elastin, and fibrinogen (Figure 3). When cells were pre-incubated with anti-rHsp60 antibodies, and then used in similar adhesion experiments, there was a significant reduction in the cell ability to adhere to laminin, elastin, fibrinogen, and fibronectin (Figure 3). For the case of type-I and type-II collagen, the blocking of Hsp60 with the polyclonal antibodies did not affect the cells’ ability to adhere to these two ECM components (Figure 3). When similar experiments were performed with cells pre-incubated with anti-rPap1 antibodies, a significant reduction was observed in the cells’ ability to adhere to all the ECM proteins tested, including type-I and type-II collagen (Figure 3). This reduction was similar to that observed for cells pre-incubated with anti-rHsp60 antibodies, but was significantly different when compared to the case of adhesion to fibronectin (Figure 3). When cells were pre-incubated with antibodies against both recombinant proteins, the adhesion to laminin, elastin, and fibronectin was significantly reduced when compared to cells pre-incubated with only one antibody, suggesting both proteins contributed to the adhesion of these ECM components (Figure 3). For the case of fibrinogen, the reduction was not significantly different from the systems with cells pre-incubated with only one antibody (*p* = 0.14, and 0.11 when compared to cells pre-incubated with anti-rHsp60 or anti-Pap1, respectively). For the case of collagens, adhesion was not significantly affected, confirming the lack of participation of Hsp60 in the adhesion of these ECM components (Figure 3). Control experiments with pre-immune sera gave adhesion profiles similar to those with yeast-like cells without incubation with antibodies (Figure 3), and controls without secondary antibodies gave no detectable signals (data not shown). Collectively, these data suggest that both Hsp60 and Pap1 contribute to *S. schenckii* adhesion to ECM, with differential adhesion properties.

**Figure 4:**
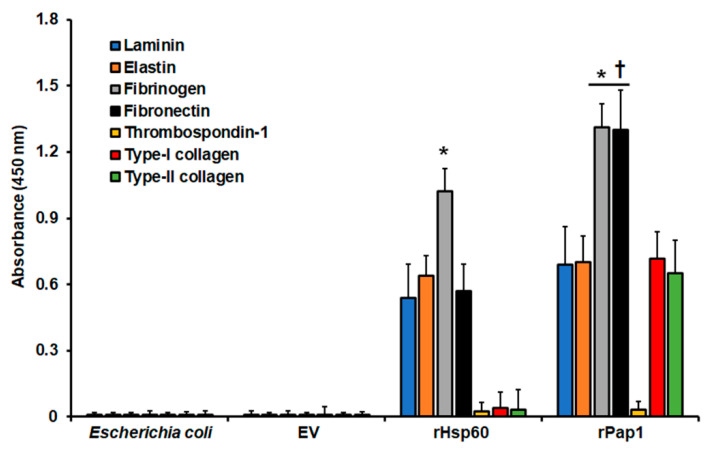
Adhesion of recombinant Hsp60 and Pap1 to immobilized extracellular matrix proteins. The extracellular matrix proteins were immobilized in 96-well plates and used in ELISA-based analyses. Cell homogenates from nontransformated bacteria (*Escherichia coli*) and bacteria transformed with the empty vector (EV) expressing rHsp60 (rHsp60) or rPap1 (rPap1) were incubated with the immobilized extracellular matrix proteins, and the protein–protein adhesion detected with the specific polyclonal anti-rHsp60 or anti-rPap1 and peroxidase-conjugated anti-rabbit IgG. Results are means ± SD of three independent experiments performed by duplicate. * *p* < 0.05 when compared to the other immobilized proteins. ^†^ *p* < 0.05 when compared to cell homogenates containing rHsp60.

Next, to confirm that both recombinant Hsp60 and Pap1 possess adhesive properties, we performed similar experiments with immobilized ECM components but, in this case, with homogenates from cells expressing the recombinant proteins. Since we detected the adhesion of these proteins to the immobilized ECM proteins using the specific polyclonal antibodies, results were unlikely affected by the endogenous adhesins present in the bacterial system. The homogenates containing rHsp60 showed a strong ability to adhere to fibrinogen, but also adhesion to laminin, elastin, and fibronectin (Figure 4). However, adhesion to thrombospondin-1 and type-I and type-II collagens was minimal, closer to the detection threshold (Figure 4). For the case of cell homogenates containing rPap1, these showed adhesion to all the ECM components, except for thrombospondin-1 (Figure 4). The homogenates showed the highest adhesion to fibrinogen and fibronectin (Figure 4). Control experiments using cell homogenates from nontransformed cells or cells transformed with the empty expression vector gave no significant readings (Figure 4). Moreover, the ability of homogenates containing rPap1 to adhere to laminin and elastin was similar to that observed for homogenates containing rHsp60, but for both fibrinogen and fibronectin was significantly higher (Figure 4). Similarly, experiments without secondary antibodies or with pre-immune sera instead of polyclonal anti-rHsp60 or anti-rPap1 gave no detectable signals (data not shown). Finally, experiments where cell homogenates were incubated at 100 °C for 15 min, to denature proteins, gave no detectable signals (not shown). Collectively, these results suggest that rHsp60 and rPap1 are adhesins that bind differently to ECM components.

### 3.4. Hsp60 and Pap1 Contribute to Sporothrix schenckii Virulence

The role of both Hsp60 and Pap1 in *S. schenckii* virulence was next assessed in *G. mellonella* larvae. We decided to use this system because it replicated the fungal virulence observed in the murine model and allowed us to include more animals per group than the mammalian system, strengthening the statistical power [7,68,69]. We first analyzed the inoculation effect of recombinant proteins on insect immune effectors. After the inoculation of different doses of both recombinant proteins, no animal died in the 2-week observation period (data not shown). Hemocyte concentration, melanin production, and phenoloxidase activity, all measured in the insect hemolymph, are immunological parameters that were previously demonstrated to change in response to fungal or bacteria stimuli [7,56,61,62,70]. Thus, we measured these immunological parameters in animals inoculated with recombinant proteins 5 days after the inoculation. Doses of 10 and 20 µg rHsp60 did not change these three parameters, but inoculation of 40 µg rHsp60 positively affected hemocyte count, melanin production, and phenoloxidase activity (Table 2). The levels of these three parameters were significantly increased when animals were inoculated with 80 µg rHsp60, and these figures did not increase upon inoculation of 160 µg rHsp60 (Table 2). When experiments were performed with rPap1, a similar result was obtained with 10 µg recombinant protein, i.e., no significant changes in the three parameters analyzed (Table 2). However, with doses ranging from 20 to 80 µg rPap1, a dose-dependent increment in the levels of hemocytes, melanin, and phenoloxidase were observed in the *G. mellonella* hemolymph (Table 2). Animals inoculated with 80 or 160 µg recombinant protein showed similar hemocyte, melanin, and phenol oxidase levels (Table 2).

**Figure 5:**
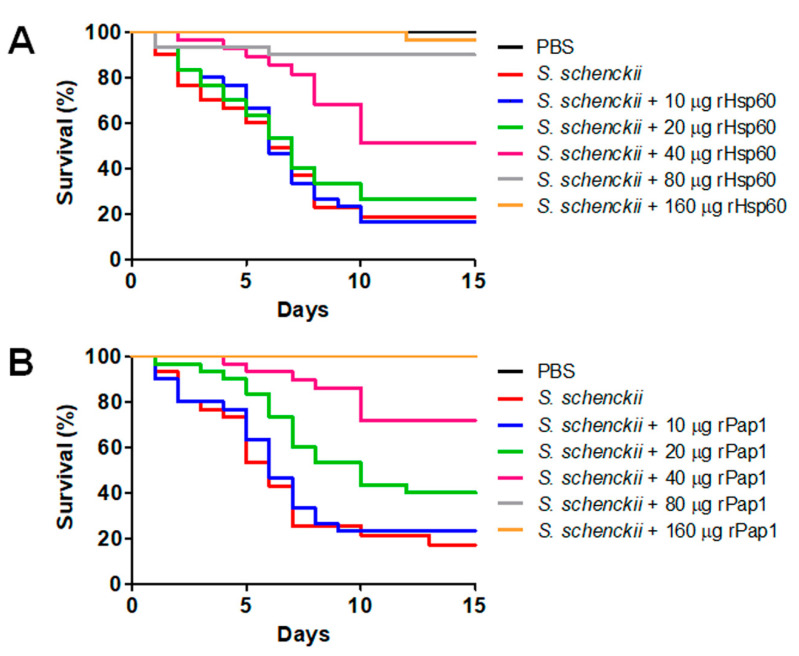
Effect of recombinant Hsp60 and Pap1 on the *Galleria mellonella* killed by *Sporothrix schenckii. G. mellonella* larvae were inoculated with either phosphate-buffered saline (PBS) or the indicated amount of rHsp60 (**A**) or rPap1 (**B**) and incubated for 5 days at 37 °C. Then, a lethal *S. schenckii* inoculum of 1 × 10^5^ yeast-like cells was injected into larvae, which were kept at 37 °C, and mortality was recorded daily. PBS, control group inoculated and challenged with PBS. *S. schenckii*, animal group inoculated with PBS and challenged with 1 × 10^5^ yeast-like cells.

Next, we assessed the effect of the recombinant protein inoculation before a lethal challenge of *S. schenckii* yeast-like cells. We inoculated the animal groups with the different doses of recombinant proteins, and 5 days later, applied an inoculation of 1 × 10^5^ yeast-like cells, a dose that kills most of the animal population in the 2-week observation period [60]. Cells inoculated only with PBS, then challenged with the fungal cells, showed a typical kill curve, with a median survival of 6.0 ± 1.0 days and 83.3 ± 3.0% population death during the observation period, which was similar to data reported previously for this fungal strain [7,30,56,60,68,69]. The inoculation of 10 or 20 µg rHsp60 did not significantly modify the kill curve, but inoculation of 40 µg rHsp60 significantly changed the kill curve with a median survival of more than 15 days and only 53.0 ± 6.0% animal death at the end of the observation period (Figure 5A). Inoculation of 80 µg or 160 µg rHsp60 before the challenge with the fungal cells significantly affected *S. schenckii* cells’ ability to kill the animal population, with only three and one death observed, respectively (Figure 5A). When compared, both curves were not significantly different (*p* = 0.53). Following the same experimental setting, the inoculation of 10 µg rPap1 and the subsequent challenge with *S. schenckii* cells generated a kill curve similar to that observed with animals inoculated with PBS then challenged with yeast-like cells (Figure 5B), with a mean survival of 6.0 ± 0.5 days and 79.3 ± 6.0% animal population death at the end of the observation period. However, inoculation of 20 µg rPap1 significantly changed the kill curve (*p* < 0.05), with a median survival of 10.0 ± 1.5 days and 60.0 ± 2.0% dead animals (Figure 5B). The inoculation of 40 µg rPap1 decreased animal mortality even further, with a median survival of more than 15 days and a mortality of 24.0 ± 7.0% (Figure 5B). The *S. schenckii* cells were incapable of killing larvae when either 80 or 160 µg rPap1 were previously inoculated in the animal groups (Figure 5B). When colony-forming units were calculated in the animal groups pre-inoculated with rHsp60 and challenged with yeast-like cells, we did not observe changes in the fungal burden when 10 or 20 µg rHsp60 were inoculated or when compared to animals inoculated only with PBS, but the inoculation of 40 µg rHps60 5 days before the fungal challenge significantly reduced the CFUs, and this was even more evident when 80 or 160 µg recombinant protein were pre-inoculated (Table 3). The immunological parameters analyzed, named hemocytes concentration, phenoloxidase activity, and melanin, were positively affected only with the pre-inoculation of 80 or 160 µg rHsp60 (Table 3). For the case of the pre-inoculation of rPap1, 10 µg did not modify the parameters under analysis (Table 3), but a dose-dependent reduction in the CFUs was observed with pre-inoculation of rPap1 in the range of 20 to 80 µg; although figures calculated with a dose of 160 µg were similar to those obtained with 80 µg (Table 3). The immunological parameters analyzed were significantly incremented when animal groups were pre-inoculated with 40, 80, or 160 µg rPap1, but in this case, a dose-dependent behavior was not observed (Table 3).

**Figure 6:**
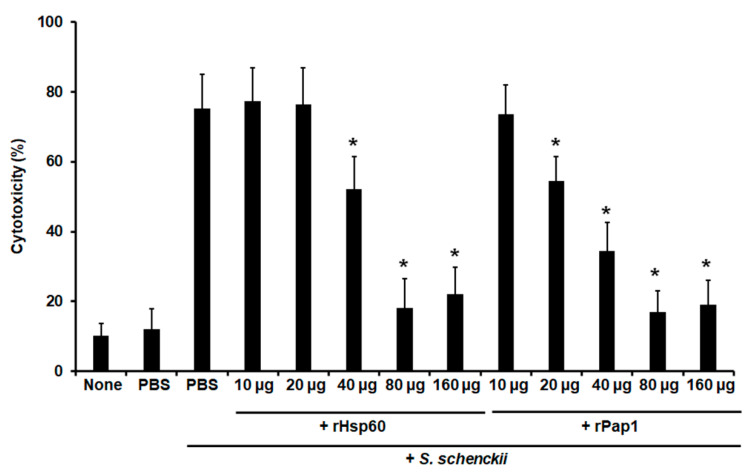
Effect of recombinant Hsp60 and Pap1 on the cytotoxicity associated to *Galleria mellonella* killed by *Sporothrix schenckii. G. mellonella* larvae were inoculated with either phosphate-buffered saline (PBS) or the indicated amount of rHsp60 or rPap1 and incubated for 5 days at 37 °C. A lethal *S. schenckii* inoculum of 1 × 10^5^ yeast-like cells was injected into larvae and incubated at 37 °C for 24 h, and the cell-free lactate dehydrogenase activity was quantified in the hemolymph. PBS, control group inoculated and challenged with PBS. PBS + *S. schenckii*, animal group inoculated with PBS and challenged with 1 × 10^5^ yeast-like cells. None, noninoculated animal group. Results are means ± SD of three independent experiments performed by duplicate. * *p* < 0.05 when compared to PBS + *S. schenckii.*

Moreover, cell-free LDH activity was measured as an indicator of cellular cytotoxicity, as previously reported [7,64]. Animal groups pre-incubated with 10 or 20 µg Hsp60 showed similar cytotoxicity levels to the control group pre-inoculated with PBS and challenged with yeast-like cells (Figure 6), but pre-inoculation of 40 µg Hsp60 significantly reduced cytotoxicity levels, and these were even more diminished with pre-inoculation of either 80 or 160 µg Hsp60 (Figure 6). When rPap1 was used in the pre-inoculation stage, 10 µg did not affect cytotoxicity levels, but 20 µg significantly reduced the values of free LDH, and those were even more reduced with pre-inoculation of either 40 or 80 µg rPap1 (Figure 6). The cytotoxicity associated with the pre-incubation of 160 µg rPap1 was similar to that obtained with 80 µg Pap1 (Figure 6).

**Figure 7:**
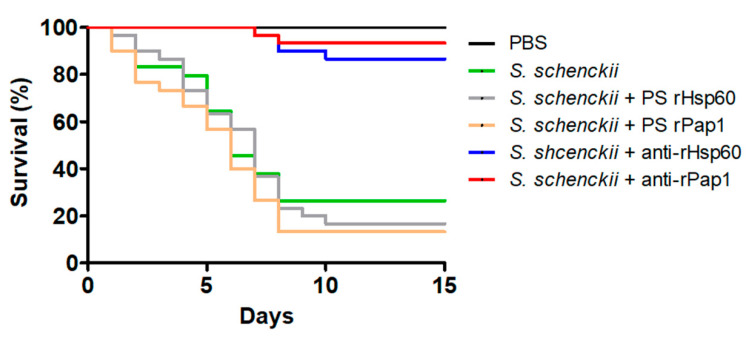
Effect of anti-recombinant Hsp60 and Pap1 antibodies on the *Galleria mellonella* killed by *Sporothrix schenckii. G. mellonella* larvae were inoculated with either phosphate-buffered saline (PBS), 1 × 10^5^ yeast-like cells (*S. schenckii*), or fungal cells pre-incubated with pre-immune sera (PS) of hosts where anti-rHsp60 or anti-Pap1 antibodies were produced or pre-incubated with polyclonal anti-rHsp60 or anti-rPap1 antibodies (*S. schenckii* + rHps60 and *S. schenckii* + rPap1, respectively) and kept at 37 °C, and mortality was recorded daily. Pre-immune sera and polyclonal antibodies were used at a working dilution of 1:2000.

**Figure 8:**
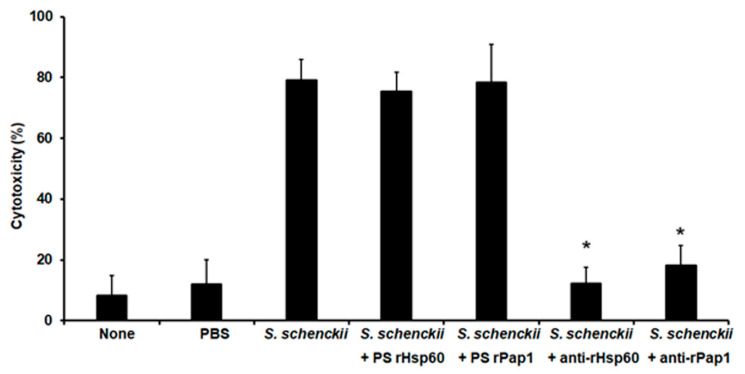
Effect of anti-recombinant Hsp60 and Pap1 antibodies on the cytotoxicity associated with *Galleria mellonella* killed by *Sporothrix schenckii. G. mellonella* larvae were inoculated with phosphate-buffered saline (PBS), 1 × 10^5^ yeast-like cells (*S. schenckii*), or fungal cells pre-incubated with pre-immune sera (PS) of hosts where anti-rHsp60 or anti-Pap1 antibodies were produced or pre-incubated with polyclonal anti-rHsp60 or anti-rPap1 antibodies (*S. schenckii* + rHps60 and *S. schenckii* + rPap1, respectively), then incubated at 37 °C for 24 h, and the cell-free lactate dehydrogenase activity was quantified in the hemolymph. None, noninoculated animal group. Results are means ± SD of three independent experiments performed by duplicate. * *p* < 0.05 when compared to the animal group inoculated with *S. schenckii* with no pre-incubation.

Next, we assessed the potential of the polyclonal anti-rHsp60 and anti-rPap1 to protect against experimental systemic sporotrichosis. The pre-immune sera or the polyclonal antibodies did not affect the animal viability or immunological parameter in the 2-week observation period (data not shown). When fungal cells were pre-incubated with polyclonal anti-Hsp60 and these cells were used to challenge *G. mellonella* larvae, most of the animal population survived to the observation period with median survivals of more than 15 days (Figure 7). Similarly, cells pre-incubated with anti-rPap1 antibodies showed attenuation in the ability to kill larvae, and this was not significantly different when compared with that observed with anti-rHsp60-treated cells (Figure 7). Animal groups inoculated with yeast-like cells or fungal cells pre-incubated with control sera showed no significant differences (Figure 7). Additionally, no animal death was observed in the control group inoculated with PBS (Figure 7). The fungal burden showed a correlation with the effect of polyclonal antibodies on the ability of *S. schenckii* cells to kill animals. Similar fungal burdens were observed in animals inoculated with yeast-like cells or fungal cells pre-incubated with pre-immune sera (Table 4), and CFUs were significantly reduced in the animal groups challenged with yeast-like cells pre-incubated with either anti-rHsp60 or anti-rPap1 (Table 4). Both cellular and humoral immune effectors, named hemocyte levels and phenoloxidase and melanin production, respectively, showed a similar trend in the animal group analyzed. These three immune effectors were high and similar in animal groups inoculated with *S. schenckii* yeast-like cells and fungal cells pre-incubated with pre-immune sera against rHsp60 or rPap1 (Table 4). However, animal groups inoculated with yeast-like cells pre-incubated with either anti-rHps60 or anti-rPap1 showed a significant reduction in the three immunological parameters (Table 4). When cytotoxicity was analyzed in these animal groups, this was significantly lower and similar in larvae inoculated with fungal cells pre-incubated with anti-rHsp60 or anti-rPap1 antibodies, when compared to the free LDH activity quantified in the hemolymph of animals inoculated with yeast-like cells with no pre-incubation step or pre-incubated with the pre-immune sera (Figure 8). The control group inoculated only with PBS showed low and similar cytotoxicity levels to those of noninoculated animals (Figure 8).

Collectively, these data strongly suggest that Hsp60 and Pap1 are proteins that contribute to the *S. schenckii*’s ability to kill *G. mellonella* larvae.

## 4. Discussion

The fungal cell wall is an attractive place to find new targets for antifungal drugs because the molecular nature of its compounds is different from the proteins, polysaccharides, and oligosaccharides found in the mammalian host and because some of these are ubiquitous molecules found in the fungal cell wall, such as glucans and chitin, providing the advantage of having wide-range targets. The *S. schenckii* cell wall has a unique component that confers the fungus immunogenicity and specific epitopes, the PRM [20].

Although PRM is a thoroughly studied *S. schenckii* cell wall component, the identities of the proteins composing this glycoconjugate remain unknown. Our mass spectrometry results showed that PRM is composed of at least 325 proteins, and the bioinformatics analysis predicted that only one out of 26 proteins listed in Table 1, named GPI-anchored cell wall beta-1,3-endoglucanase EglC, has a signal peptide and is predicted to be secreted and localized in the cell wall. It has to be acknowledged that the protocol for PRM isolation does not preclude the contamination of cell walls with intracellular proteins. However, the harsh conditions used here and the PRM enrichment by chromatographies make it unlikely that any contaminant protein could co-purify along with PRM. Moreover, the detection of proteins listed in Table 1 was constant in all the experimental replicates analyzed by mass spectrometry, and we found biological activities at least for Hsp60 and Pap1 linked to the cell wall, named adhesion properties, and an effect on host mortality upon pre-incubation of yeast-like cells with polyclonal anti-rHsp60 or anti-rPap1 antibodies, making it unlikely these proteins were intracellular contaminants. Based on this, we suggest that most of the PRM proteins might be moonlighting proteins that are transported to the cell wall via noncanonical secretory pathways [71]. A moonlighting protein is defined as a multifunctional protein that participates in more than one independent biological process, usually in different cell compartments [72], and since most of the identified proteins found in the PRM have putative housekeeping intracellular functions or participate in metabolic pathways, the PRM proteins may have a second function in the *S. schenckii* cell wall, probably participating in host–fungus interaction. Moonlighting proteins in the cell wall of other pathogenic fungi participate in cell adhesion to different host components. In *Candida albicans*, Ssa1 and Ssa2, members of the Hsp70 family, bind to the host-cell cadherins [73], enolase binds to plasminogen [74], glyceraldehyde-3-phosphate dehydrogenase 1 and 2 bind to fibronectin, laminin, and complement regulators [32,75], and phosphate-glycerate mutase bind to plasminogen and complement regulators [76,77], among others [35]. In *H. capsulatum,* Hsp60 is the major ligand to the complement receptor 3 (CR3; CD11b/CD18) in alveolar macrophages [37]; and in *Cryptococcus neoformans,* phosphoglycerate kinase and fructose biphosphate aldolase were found to bind to plasminogen [78]. The presence of moonlighting proteins in the *S. schenckii* cell wall was previously suggested. The cell wall proteomic analysis of cells responding to menadione, an oxidative agent, found 13 proteins that could be involved in the fungal oxidative stress response, some of which were already reported as proteins with dual localization and functions in *Candida* spp. [79]. Another study suggested that the proteins Hsp60, chaperonin GroEL, elongation factor 1β, and mitochondrial peroxiredoxin participate in oxidative stress, playing a role in *S. schenckii* survival within the host [80]. However, these studies did not confirm the proteins’ second function nor localization within the *S. schenckii* cell.

Out of the PRM proteins found, two stood out due to their high coverage and Andromeda scores, Hsp60 and the previously uncharacterized Pap1. Heat shock proteins are highly conserved in all living organisms and upregulated in response to different stress conditions, playing important roles in cell homeostasis [81]. Their main function is proper protein folding, unfolding, and translocation and the assembly and disassembly of protein complexes. Members of the Hsp60 family, also called mitochondrial chaperones, assist in the refolding of denatured proteins under stress conditions, as well as in the folding of newly synthesized proteins and as a transporter of proteins targeted to different cellular compartments, and are preferentially found in the mitochondria and cytoplasm [82,83]. In addition to these canonical functions, members of the Hsp60 family of proteins were also reported to be highly immunogenic in different pathogenic organisms, including *H. capsulatum* [36], *P. brasiliensis,* and *Paracoccidioides lutzii* [84,85], and were also localized within the fungal cell wall [38].

The expression patterns of *S. schenckii HSP60*, named upregulation in yeast-like cells and fungal cells co-incubated with human cells, agreed with previous observations in *Aspergillus fumigatus*, *Aspergillus terreus*, *Scedosporium apiospemum*, *C. albicans*, *Penicillium chrysogenum*, *P. brasiliensis,* and *Trichophyton mentagrophytes*, where exposure to high temperatures caused an *HSP60* temperature-dependent upregulation [38,86].

For the case of Pap1, as mentioned, this protein has putative homologs in *O. piceae* and *G. clavigera*, and in the former, the protein was predicted to be at the cell surface. In addition, these two proteins, along with Pap1, were predicted to contain disordered domains and were considered intrinsically disordered proteins (https://iupred2a.elte.hu; accessed on 5 October 2018) [50]. These kinds of proteins are characterized by the lack of a well-defined folding and structure under physiological conditions, and thus, are considered out of the structure–function paradigm, and their function cannot be predicted by structural motifs [87,88]. Some of the functions already demonstrated for these proteins include molecular recognition and binding to other molecules, which are consequences of the direct transition from ordered to disordered flexible regions [88]. Interestingly, many moonlighting proteins are also intrinsically disordered proteins and participate in protein–protein interactions [89]. We observed that most of the main PRM proteins given in Table 1 were predicted to be long disordered proteins with scores higher than 0.5 (data not shown). These proteins are heat shock 70 kDa protein 1/8, elongation factor 1-alpha, GPI-anchored cell wall beta-1,3-endoglucanase EglC, large subunit ribosomal protein LP2, AMPK1_CBM domain-containing protein, molecular chaperone DnaK, glucose-repressible protein, peptidyl-prolyl cis-trans isomerase, large subunit ribosomal protein LP1, sphingolipid long-chain base-responsive protein, acetyltransferase component of pyruvate dehydrogenase complex, mismatched base pair and cruciform DNA recognition protein, aconitate hydratase mitochondrial, cytochrome C oxidase subunit 5b, actin beta/gamma 1, and the uncharacterized proteins SPSK_04236, SPSK_05930, SPSK_01041, SPSK_02764, and SPSK_06559. The proteins glyceraldehyde-3-phosphate dehydrogenase, ATP synthase subunit beta, and 2-phosphoglycerate dehydratase were predicted to be short disordered, while Hsp60 and aldehyde dehydrogenase (NAD+) were predicted to be ordered/structured proteins (data not shown). These observations agreed with our suggestion that these proteins are likely to be moonlight proteins. Moreover, the *PAP1* expression indicated is upregulated in yeast-like cells and when interacting with host cells, adding further evidence to suggest it could have a role during the *S. schenckii*–host interaction.

The findings presented here showed Hsp60 and Pap1 are PRM members and are likely moonlighting proteins that participate in the adhesion of the ECM to components. In agreement, a 62 kDa protein isolated from the *H. capsulatum* cell wall, named HIS-62/Hsp60, was shown to play important roles during the interaction with the host, inducing T-cell activation and the stimulation of a protective immune response [36]. In addition, it contributes to fungal virulence due to its adhesive properties [37]. A similar observation was also reported for *P. brasiliensis* Hsp60 [66,90]. The fact that Pap1 was predicted to be an intrinsically disordered protein may offer a possible explanation for the wider ability to bind ECM components when compared to Hsp60.

Gp70, besides being a highly immunogenic protein, is an *S. schenckii* adhesin that mediates binding to the host dermal matrix and fibronectin [4,91,92]. However, unlike Hsp60 and Pap1, Gp70 is a typical cell wall protein with a signal peptide [93] and thus is likely to be transported by conventional secretion systems. Other putative adhesins for fibronectin, laminin, and type-II collagen in the *S. schenckii* cell wall have been described, with variations among strains and morphologies [59,92,94,95]. However, the identity of the proteins with those adhesive properties has not been established.

When inoculated to *G. mellonella* larvae before challenging with a lethal dose of *S. schenckii* cells, both rHsp60 and rPap1 conferred protection against the experimental infection, and this observation resembles the immunological priming already reported in invertebrates, a response similar to the immunological memory found in the mammalian host and other vertebrates [96]. In *G. mellonella*, immunological priming was stimulated using bacteria, bacterial lipopolysaccharide, and *S. schenckii* recombinant proteins [56,61,97,98]. It was recently reported that a recombinant version of the *S. schenckii* cell wall protein Gp70 stimulated immunological priming in *S. schenckii* cells, increasing hemocyte levels and phenoloxidase activity [56]. As a consequence, increased survival rates of animals infected with *S. schenckii* cells were observed [56]. Similarly, the inoculation of either rHsp60 or rPap1 stimulated immunological priming and reduced the mortality associated with the inoculation of the fungal cells, most likely due to the immunological activation by the recombinant proteins, allowing the animals to control fungal cells. This was supported by the low fungal burden and cytotoxicity observed in the hemolymph of infected animals. The fact that a lower dose of rPap1 was sufficient to generate these observations when compared to Hsp60 could be explained by the fact that the former is an intrinsically disordered protein and this could be an advantage to stimulate immune sensors involved in the immunological priming stimulation. This hypothesis remains to be addressed though. Our results here pave the way to assess the relevance of these proteins during the interaction with the mammalian host, such as the murine model of experimental sporotrichosis. One limitation we have to acknowledge about our experimental design is that it did not assess the contribution of the glycan moieties modifying Hsp60 and Pap1 during interaction with the host. However, nonglycosylated versions of the Gp70 are capable of stimulating a protective immune response in the murine model of infection caused by *S. brasiliensis* and *S. globosa* [99,100].

The clinically relevant species that cause sporotrichosis, *S. schenckii*, *S. brasiliensis,* and *S. globosa* were reported to have different virulence profiles [2]. *S. brasiliensis* was described as the most virulent species, and was associated with severe clinical forms of the mycosis, followed by *S. schenckii* with moderate virulence, and *S. globosa*, considered a low-virulence species [2]. These different virulence profiles can be related to the virulence factors that the different species have, such as adhesins. Since the Hsp60 is a highly conserved protein, it was found in the three *Sporothrix* species, while the Pap1 was found to be only present in *S. schenckii* and *S. brasiliensis* but not in *S. globosa*. This finding might suggest a correlation between virulence and the presence of this protein in the fungus, attributing Pap1 an important role during the *S. schenckii*–host interaction.

It has been widely reported that the administration of antibodies can alter the course of experimental mycoses, such as paracoccidioidomycosis, histoplasmosis, and even sporotrichosis. When monoclonal antibodies against the *H. capsulatum* Hsp60 were used during the interaction of *P. lutzii* with immune cells and on experimental infection, a protective response was observed [85], showing cross-reactivity of antibodies and the high degree of similarity of the protein from both fungal species. The antibodies bound to the yeast cells and promoted phagocytosis by macrophages in vitro, and treatment of mice with the antibodies before infection reduced the fungal burden [85]. In addition, when these monoclonal antibodies were used in a mouse model of histoplasmosis, a decreased in intracellular fungal survival and increased phagocytosis and phagolysosomal fusion of macrophages was observed, with prolonged animal survival and reduction of the organ fungal burden and tissue inflammation [101]. Moreover, the use of a chimeric IgG mouse-human monoclonal antibody against Hsp60 altered the course of experimental histoplasmosis and paracoccidioidomycosis, increasing phagolysosomal fusion and enhancement of yeast phagocytosis by macrophages in vitro and reducing the fungal burden in a murine model of intranasal infection with *H. capsulatum* [102]. Our results are in line with these observations, since the polyclonal antibodies anti-rHsp60 and anti-rPap1 were capable of protecting against systemic sporotrichosis, suggesting that both Hsp60 and Pap1 are adhesins that have a major contribution to *S. schenckii* virulence.

In conclusion, our results indicate that the peptidic component of PRM is a heterogenous group of proteins, which are likely to be moonlighting and highly disordered. Among them, Hsp60 and Pap1 were found to be adhesins that bind several ECM components and were capable of inducing immunological priming. Blocking either Hsp60 or Pap1 with polyclonal anti-rHsp60 or anti-Pap1 antibodies indicated that they are important players in the *S. schenckii*–host interaction.

## Figures and Tables

**Table 1:** Main identified proteins in peptidorhamnomannan by capillary liquid-chromatography-electrospray ionization-quadrupole time-of-flight mass spectrometry.

Protein	Gene	Coverage (%)	*q*-Value	Andromeda Score
Chaperonin GroEL-like protein/heat shock protein 60	SPSK_01586	48.9	0	323
Heat shock 70 kDa protein 1/8	SPSK_08625	47.6	0	323
Uncharacterized protein	SPSK_04236	22.2	0	323
Uncharacterized protein	SPSK_00848	21.3	0	323
Uncharacterized protein	SPSK_05930	19.8	0	323
Glyceraldehyde-3-phosphate dehydrogenase	SPSK_00294	38.3	0	322
ATP synthase subunit beta	SPSK_01537	54.7	0	307
Elongation factor 1-alpha	SPSK_06026	33.5	0	291
GPI-anchored cell wall beta-1,3-endoglucanase EglC	SPSK_01694	21.2	0	260
Aldehyde dehydrogenase (NAD+)	SPSK_00262	28.4	0	244
Large subunit ribosomal protein LP2	SPSK_03751	34.2	0	218
Uncharacterized protein	SPSK_01041	23.4	0	217
AMPK1_CBM domain-containing protein	SPSK_08400	27.6	0	197
Molecular chaperone DnaK	SPSK_03148	22.9	0	187
Glucose-repressible protein	SPSK_01322	64.8	0	182
Peptidyl-prolyl cis-trans isomerase	SPSK_01963	24.2	0	172
Uncharacterized protein	SPSK_02764	50.3	0	158
Large subunit ribosomal protein LP1	SPSK_05488	43.6	0	149
Sphingolipid long chain base-responsive protein	SPSK_05417	21.7	0	116
Acetyltransferase component of pyruvate dehydrogenase complex	SPSK_07465	14.7	0	115
Mismatched base pair and cruciform DNA recognition protein	SPSK_05604	28.3	0	113
Aconitate hydratase, mitochondrial	SPSK_00414	13.8	0	110
Uncharacterized protein	SPSK_06559	32.6	0	110
Cytochrome c oxidase subunit 5b	SPSK_03566	29.4	0	110
2-phosphoglycerate dehydratase	SPSK_03292	20.5	0	106
Actin beta/gamma 1	SPSK_00108	15	0	104

**Table 2:** Hemocyte count, phenoloxidase, and melanin production in *Galleria mellonella* larvae inoculated with either recombinant Hsp60 or Pap1.

Inoculated with	Hemocytes (×10^6^) mL^−1 a^	Phenoloxidase ^b^	Melanin ^c^
None	3.3 ± 0.4	0.5 ± 0.3	0.7 ± 0.4
PBS ^d^	3.1 ± 0.7	0.8 ± 0.5	1.1 ± 0.2
10 µg rHsp60	2.9 ± 0.6	0.7 ± 0.4	1.3 ± 0.5
20 µg rHsp60	3.2 ± 0.5	1.0 ± 0.3	1.0 ± 0.3
40 µg rHsp60	6.9 ± 0.8 *	1.9 ± 0.6 *	2.8 ± 0.4 *
80 µg rHsp60	9.8 ± 0.4 * ^†^	2.7 ± 0.6 * ^†^	4.2 ± 0.8 * ^†^
160 µg rHsp60	10.1 ± 0.7 * ^†^	3.1 ± 0.7 * ^†^	3.8 ± 0.4 * ^†^
10 µg rPap1	3.3 ± 0.8	0.9 ± 0.2	1.1 ± 0.2
20 µg rPap1	5.3 ± 0.6 *	1.6 ± 0.4 *	2.1 ± 0.4 *
40 µg rPap1	8.2 ± 0.5 * ^†^	2.4 ± 0.4 * ^†^	3.2 ± 0.4 * ^†^
80 µg rPap1	10.2 ± 0.6 * ^†^	3.4 ± 0.3 * ^†^	4.3 ± 0.6 * ^†^
160 µg rPap1	9.8 ± 0.6 * ^†^	3.3 ± 0.5 * ^†^	4.0 ± 0.5 * ^†^

^a^ Larvae were injected with the indicated inoculum and monitored for 15 days, then hemolymph was collected and used to calculate hemocyte concentration. ^b^ Defined as the Δ_490nm_ per min per μg protein. ^c^ Defined as the absorbance at 405 nm of the cell-free hemolymph. ^d^ Animal control group inoculated with PBS. * *p* < 0.05 when compared with the values obtained with animals inoculated with PBS. ^†^ *p* < 0.05 when compared with values obtained with different concentrations of the same recombinant protein.

**Table 3:** Colony-forming units, hemocyte counts, phenoloxidase, and melanin production in *Galleria mellonella* larvae pre-inoculated with either recombinant Hsp60 or Pap1 and challenged with *Sporothrix schenckii* yeast-like cells.

Inoculated with	Colony-Forming Units (×10^5^) ^a^	Hemocytes (×10^6^) mL^−1 a^	Phenoloxidase ^b^	Melanin ^c^
None	0.0 ± 0.0	3.6 ± 0.2	0.4 ± 0.4	0.5 ± 0.3
PBS ^d^	0.0 ± 0.0	3.3 ± 0.3	0.9 ± 0.3	1.2 ± 0.4
PBS + *S. schenckii*	2.4 ± 0.4	8.0 ± 0.6	3.2 ± 0.6	3.5 ± 0.6
10 µg rHsp60 + *S. schenckii*	2.7 ± 0.3	7.6 ± 0.5	3.7 ± 0.3	3.3 ± 0.3
20 µg rHsp60 + *S. schenckii*	2.3 ± 0.4	8.1 ± 0.3	3.2 ± 0.5	3.3 ± 0.3
40 µg rHsp60 *S. schenckii*	1.3 ± 0.2 *	7.2 ± 0.5	2.6 ± 0.5	3.0 ± 0.2
80 µg rHsp60 + *S. schenckii*	0.4 ± 0.2 *	10.5 ± 0.5 *	4.4 ± 0.4 *	4.4 ± 0.3 *
160 µg rHsp6 + *S. schenckii*	0.2 ± 0.2 *	10.3 ± 0.3 *	4.6 ± 0.2 *	4.6 ± 0.5 *
10 µg rPap1 + *S. schenckii*	2.7 ± 0.5	7.3 ± 0.7	2.9 ± 0.5	3.1 ± 0.5
20 µg rPap1 + *S. schenckii*	1.9 ± 0.3 *	7.7 ± 0.6	3.3 ± 0.3	3.2 ± 0.5
40 µg rPap1 + *S. schenckii*	1.1 ± 0.2 *	10.2 ± 0.3 *	4.4 ± 0.3 *	4.1 ± 0.3 *
80 µg rPap1 + *S. schenckii*	0.3 ± 0.2 *	10.6 ± 0.4 *	4.6 ± 0.5 *	4.7 ± 0.5 *
160 µg rPap1 + *S. schenckii*	0.2 ± 0.1 *	10.8 ± 0.8 *	4.5 ± 0.3 *	4.5 ± 0.4 *

^a^ Larvae were injected with the indicated inoculum and monitored for 15 days, then hemolymph was collected and used to calculate colony-forming units and hemocyte concentration. ^b^ Defined as the Δ_490nm_ per min per μg protein. ^c^ Defined as the absorbance at 405 nm of the cell-free hemolymph. ^d^ Animal control group inoculated with PBS. * *p* < 0.05 when compared with the values obtained with animals pre-inoculated with PBS and challenged with *S. schenckii* yeast-like cells.

**Table 4:** Colony-forming units, hemocyte count, phenoloxidase, and melanin production in *Galleria mellonella* larvae challenged with *Sporothrix schenckii* yeast-like cells.

Inoculated with	Colony-Forming Units (×10^5^) ^a^	Hemocytes (×10^6^) mL^−1 a^	Phenoloxidase ^b^	Melanin ^c^
None	0.0 ± 0.0	3.8 ± 0.4	0.3 ± 0.2	0.4 ± 0.2
PBS ^d^	0.0 ± 0.0	3.9 ± 0.5	0.7 ± 0.5	0.9 ± 0.6
*S. schenckii*	2.3 ± 0.5	8.0 ± 0.3	2.9 ± 0.4	3.9 ± 0.3
*S. schenckii* + PS rHsp60	2.8 ± 0.6	8.2 ± 0.4	3.1 ± 0.5	3.7 ± 0.6
*S. schenckii* + PS rPap1	2.6 ± 0.6	7.8 ± 0.3	3.4 ± 0.3	3.9 ± 0.5
*S. schenckii* + anti-rHsp60	0.9 ± 0.5 *	4.3 ± 0.6 *	0.7 ± 0.2 *	0.8 ± 0.5 *
*S. schenckii* + anti-rPap1	0.5 ± 0.4 *	3.5 ± 0.7 *	0.6 ± 0.6 *	0.5 ± 0.4 *

^a^ Larvae were injected with the indicated inoculum and monitored for 15 days, then hemolymph was collected and used to calculate colony-forming units and hemocyte concentration. ^b^ Defined as the Δ_490nm_ per min per μg protein. ^c^ Defined as the absorbance at 405 nm of the cell-free hemolymph. ^d^ Animal control group inoculated with PBS. * *p* < 0.05 when compared with the values obtained with animals inoculated with *S. schenckii* cells. PS, pre-immune sera.

## Supplementary Materials

The following are available online at https://www.mdpi.com/article/10.3390/jof7110960/s1, Table S1: Proteins identified in peptidorhamnomannan by capillary liquid-chromatography-electrospray ionization-quadrupole time-of-flight mass spectrometry
