# Supplementary material for: The Heat Shock Protein 60 and Pap1 Participate in the Sporothrix schenckii-Host Interaction
Source: J Fungi (Basel). 2021 Nov 12;7(11):960. doi: 10.3390/jof7110960 (PMC8620177; doi:10.3390/jof7110960)
Supplement: Supplementary file 1 [file jof-07-00960-s001.zip › jof-1450343-supplementary.pdf]

Table S1. Proteins identified in peptidorhamnomannan by capillary liquid-chromatography-electrospray ionization-quadrupole time-of-flight mass spectrometry.

Protein	Gene	Coverage [%]	*q*-value	Andromeda score
Chaperonin GroEL-like protein/heat shock protein 60	SPSK_01586	48.9	0	323.31
Heat shock 70kDa protein 1/8	SPSK_08625	47.6	0	323.31
Uncharacterized protein	SPSK_04236	22.2	0	323.31
Uncharacterized protein	SPSK_00848	21.3	0	323.31
Uncharacterized protein	SPSK_05930	19.8	0	323.31
Glyceraldehyde-3-phosphate dehydrogenase	SPSK_00294	38.3	0	321.72
ATP synthase subunit beta	SPSK_01537	54.7	0	306.73
Elongation factor 1-alpha	SPSK_06026	33.5	0	290.97
GPI-anchored cell wall beta-1,3-endoglucanase EglC	SPSK_01694	21.2	0	259.76
Aldehyde dehydrogenase (NAD+)	SPSK_00262	28.4	0	244.48
Large subunit ribosomal protein LP2	SPSK_03751	34.2	0	217.59
Uncharacterized protein	SPSK_01041	23.4	0	217.46
AMPK1_CBM domain-containing protein	SPSK_08400	27.6	0	196.68
Molecular chaperone DnaK	SPSK_03148	22.9	0	187.33
Glucose-repressible protein	SPSK_01322	64.8	0	181.87
Peptidyl-prolyl cis-trans isomerase	SPSK_01963	24.2	0	172.2
Uncharacterized protein	SPSK_02764	50.3	0	158.32
Large subunit ribosomal protein LP1	SPSK_05488	43.6	0	148.6
Sphingolipid long chain base-responsive protein	SPSK_05417	21.7	0	115.71
Acetyltransferase component of pyruvate dehydrogenase complex	SPSK_07465	14.7	0	115.07
Mismatched base pair and cruciform DNA recognition protein	SPSK_05604	28.3	0	112.78
Aconitate hydratase, mitochondrial	SPSK_00414	13.8	0	110.44
Uncharacterized protein	SPSK_06559	32.6	0	110.15
Cytochrome c oxidase subunit 5b	SPSK_03566	29.4	0	109.68
2-phosphoglycerate dehydratase	SPSK_03292	20.5	0	106.42
Actin beta/gamma 1	SPSK_00108	15	0	103.95
Malate dehydrogenase	SPSK_07596	20.6	0	97.439
Mitochondrial peroxiredoxin PRX1	SPSK_10791	35.1	0	95.088
Voltage-dependent anion channel protein 2	SPSK_08800	22.9	0	87.311
Chaperonin GroES	SPSK_08095	5.8	0	85.459
Superoxide dismutase [Cu-Zn]	SPSK_09802	33.1	0	83.381
Uncharacterized protein	SPSK_08022	42.7	0	82.907
Plasma membrane ATPase	SPSK_08388	5.4	0	81.849
Heat shock 70kDa protein 4	SPSK_00430	6.9	0	80.056

Tyrosine 3-monooxygenase/tryptophan 5-monooxygenase activation protein	SPSK_06387	22.1	0	78.489
NAD-specific glutamate dehydrogenase	SPSK_05871	7.5	0	78.431
6-phosphogluconate dehydrogenase, decarboxylating	SPSK_02374	9.2	0	78.223
Ubiquinol-cytochrome c reductase core subun	SPSK_09469	21.5	0	75.827
Ribosomal protein L19	SPSK_09543	4.5	0	74.848
Phosphoenolpyruvate carboxykinase (ATP)	SPSK_02442	15.8	0	71.238
Probable electron transfer flavoprotein subunit alpha	SPSK_09535	20.8	0	69.903
Phosphoketolase	SPSK_00433	10.5	0	69.168
Glycosidase	SPSK_03402	21.4	0	67.499
Trp repressor binding protein	SPSK_09732	21.7	0	66.76
Hydroxymethylglutaryl-CoA lyase	SPSK_00289	5.5	0	65.676
Histone H2B	SPSK_00606	40.1	0	61.529
Molecular chaperone HtpG	SPSK_08698	9.9	0	60.916
Cytochrome b5 heme-binding domain-containing protein	SPSK_05054	9.9	0	59.631
Transitional endoplasmic reticulum ATPase	SPSK_06992	8.8	0	56.343
40S ribosomal protein S1	RPS1	5.7	0	55.265
Stress-induced-phosphoprotein 1	SPSK_01598	7	0	53.903
Epimerase domain-containing protein	SPSK_00295	7.4	0	52.857
Rnp domain containing protein	SPSK_01151	19.7	0	52.299
GH16 domain-containing protein	HMPREF1624_04554	15.7	0	52.165
Methylmalonate-semialdehyde dehydrogenase (CoA acylating)	SPSK_03220	12.7	0	52.061
Phosphoglycerate kinase	SPSK_06331	15.7	0	51.592
Alcohol dehydrogenase (NADP+)	SPSK_06513	22	0	51.095
Fructose-bisphosphate aldolase	SPSK_05365	17.1	0	50.932
Large subunit ribosomal protein L5e	SPSK_04423	5.3	0	50.507
Uncharacterized protein	SPSK_00691	4.9	0	50.502
Polyadenylate-binding protein	SPSK_04034	9.1	0	50.435
Small subunit ribosomal protein S15e	SPSK_00890	41.2	0	50.019
Zinc-binding protein oxidoreductase ToxD	SPSK_07651	17.5	0	48.263
Acyl carrier protein	SPSK_02030	15.1	0	46.063
Transaldolase	SPSK_09195	21.9	0	45.1
Complement component 1 Q subcomponent-binding protein, mitochondrial	SPSK_01854	14.3	0	44.808
Glucose-6-phosphate isomerase	SPSK_08908	9.1	0	44.769
Elongation factor 2	SPSK_03018	7	0	44.368

Carboxymethylenebutenolidase	SPSK_06369	26.8	0	44.116
MSC1 protein	SPSK_07154	11.1	0	43.936
ATP synthase subunit 5, mitochondrial	SPSK_01914	20.1	0	43.371
Uncharacterized protein	SPSK_04686	66.7	0	41.715
RRM domain-containing protein	SPSK_02668	21.2	0	41.516
Elongation factor 1-beta	SPSK_09330	24	0	40.257
Inorganic diphosphatase	SPSK_06265	15.6	0	39.541
Uncharacterized protein	SPSK_08298	5.3	0	38.996
Malate dehydrogenase	SPSK_02491	17.2	0	38.922
Methionyl aminopeptidase	SPSK_07389	15.6	0	38.595
Arf-GAP domain-containing protein	SPSK_01012	22	0	38.532
Elongation factor 1-gamma	SPSK_05125	7.7	0	36.049
Homoserine dehydrogenase	SPSK_03400	12	0	36.024
GFO_IDH_MocA domain-containing protein	SPSK_09302	8.9	0	35.679
Uncharacterized protein	SPSK_09624	35.3	0	35.545
Catalase-peroxidase	katG	3.8	0	35.213
Peptidylprolyl isomerase	SPSK_07939	40.7	0	35.202
5-methyltetrahydropteroyltriglutamate--homocysteine S-methyltransferase	SPSK_04003	6.8	0	34.857
Peptidyl-prolyl isomerase D (Cyclophilin D)	SPSK_09374	16.5	0	34.653
Kynureninase	BNA5	2	0	34.086
Nascent polypeptide-associated complex subunit beta	SPSK_03653	18.1	0	34.063
ATP synthase subunit gamma	SPSK_09504	15.9	0	33.76
Heat shock 70kDa protein 1/8	SPSK_03121	8	0	33.544
Serine hydroxymethyltransferase	SPSK_00503	5.4	0	33.439
40S ribosomal protein S12	SPSK_09873	27.8	0	33.169
Acetyl-coenzyme A synthetase	SPSK_01452	2.7	0	33.001
Succinyl-CoA:3-ketoacid-coenzyme A transferase	SPSK_01628	7.3	0	32.99
Triosephosphate isomerase	SPSK_05531	18.2	0	32.681
Mitochondrial import receptor subunit TOM40	SPSK_05330	7	0	32.466
GTP-binding nuclear protein	SPSK_05396	18.1	0	32.36
2-methylcitrate dehydratase	SPSK_06155	9.5	0	32.091
Small subunit ribosomal protein S5e	SPSK_01286	19.8	0	31.899
Pyruvate decarboxylase	SPSK_00477	7.8	0	31.528
Zinc-binding protein oxidoreductase	SPSK_06436	12.8	0	31.436
Alpha,alpha-trehalose-phosphate synthase (UDP-forming)	SPSK_08430	1.1	0	30.905
Uncharacterized protein	SPSK_09399	9	0	30.27
Sorbitol/xylulose reductase Sou1	SPSK_07897	8.3	0	30.091
Small subunit ribosomal protein S3e	SPSK_07583	18.6	0	29.553

Large subunit ribosomal protein L7e	SPSK_02415	12.7	0	29.062
Catalase	SPSK_09132	10.8	0	28.754
Uncharacterized protein	SPSK_04620	24.4	0	28.584
Histone H2A	SPSK_00609	6.7	0	28.58
Uncharacterized protein	SPSK_06626	34.4	0	28.056
Dihydrolipoyllysine-residue succinyltransferase	SPSK_00516	8.3	0	28.01
Uncharacterized protein	SPSK_01246	10.8	0	27.551
Calnexin	SPSK_03189	6.5	0	27.118
Gluconolactonase	SPSK_07725	6.2	0	26.994
Catalase	SPSK_06226	1.6	0	26.919
Dihydrolipoyl dehydrogenase	SPSK_00489	7	0	26.761
HMG box-containing protein	SPSK_10101	17.5	0	26.703
Cytochrome b5	SPSK_09928	21.9	0	26.685
Endoplasmic reticulum chaperone BiP	SPSK_04019	7.5	0	26.523
ATP synthase subunit d, mitochondrial	SPSK_03589	22.5	0	26.306
Indoleamine 2,3-dioxygenase	SPSK_05234	3.9	0	26.268
Hsp98-like protein	HMPREF1624_01957	5.6	0	26.263
YCII domain-containing protein	SPSK_05532	33.3	0	25.883
Aha1_N domain-containing protein	SPSK_09566	11.5	0	25.671
Oxidoreductase, short chain dehydrogenase/reductase family superfamily	SPSK_01899	8.1	0	24.07
Large subunit ribosomal protein L12e	SPSK_04471	4.3	0	23.828
Lipase 1	SPSK_03375	6.8	0	23.746
Proteasome subunit beta	SPSK_03431	6.5	0	23.577
Sphingolipid long chain base sensory protein	SPSK_09314	10.8	0	23.07
Ribonucloprotein	SPSK_03688	21.3	0	23.011
Phospho-2-dehydro-3-deoxyheptonate aldolase	SPSK_07623	5.3	0	22.99
Peroxiredoxin (Alkyl hydroperoxide reductase subunit C)	SPSK_01912	10.4	0	22.656
Citrate synthase	SPSK_09045	9.1	0	22.452
Dynein light chain	SPSK_09627	17.6	0	21.862
Ran-binding protein 1	SPSK_06421	9.1	0	21.837
Acetyl-CoA acyltransferase	SPSK_04310	9.6	0	21.213
Citrate synthase	SPSK_02464	7	0	20.949
Alcohol dehydrogenase, propanol-preferring	SPSK_01190	6.9	0	20.479
Large subunit ribosomal protein L27Ae	SPSK_07900	6	0	20.469
Transketolase	SPSK_04927	4.7	0	20.3
Beta-glucosidase	SPSK_01027	5.2	0	19.43
Dynamin GTPase	SPSK_08578	3.1	0	19.372

Pyruvate dehydrogenase E1 component subunit beta	SPSK_06320	6.3	0	19.12
RNA-binding protein	SPSK_05667	26.1	0	19.071
Profilin	SPSK_03692	15.5	0	18.808
Ketol-acid reductoisomerase, mitochondrial	SPSK_07472	6.7	0	18.729
Formate dehydrogenase	SPSK_02444	5.5	0	18.65
1,3-beta-glucanosyltransferase	SPSK_07340	3.5	0	18.418
Uncharacterized protein	SPSK_05421	7.3	0	18.143
Uncharacterized protein	SPSK_08030	13.4	0	17.867
NADH dehydrogenase (Ubiquinone) 1 alpha subcomplex 5	SPSK_03555	7.4	0	17.805
Glycine cleavage system P protein	SPSK_02189	2.8	0	17.664
Diazepam-binding protein inhibitor (GABA receptor modulator, acyl-CoA-binding protein)	SPSK_01289	14	0	17.565
Multifunctional fusion protein	SPSK_06084	3.8	0	17.481
Uncharacterized protein	SPSK_05565	12.4	0	17.238
Protein transport protein SEC61 subunit beta	SPSK_06670	14.8	0	17.067
Large subunit ribosomal protein L31e	SPSK_04523	17.1	0	16.996
THO complex subunit 4	SPSK_01722	11.4	0	16.968
Uncharacterized protein	SPSK_04222	4	0	16.91
Uncharacterized protein	SPSK_02628	15.1	0	16.511
Prohibitin	SPSK_09626	10.4	0	16.41
TOG domain-containing protein	SPSK_00388	2.4	0	16.348
Cystathionine beta-synthase	SPSK_06205	6.5	0	16.115
UDP-N-acetylglucosamine pyrophosphorylase	SPSK_00033	2.1	0	16.013
Eukaryotic translation initiation factor 3 subunit E	INT6	5.7	0	16.011
Proteasome subunit beta	SPSK_05485	5.2	0	15.912
High mobility group protein	SPSK_00765	7.8	0	15.827
NTF2 and RRM domain protein	SPSK_05146	5.4	0	15.77
Mitochondrial ATPase inhibitor, IATP family protein	SPSK_06697	8.3	0	15.589
RNP domain protein	SPSK_00528	6.6	0	15.534
DJ-1_PfpI domain-containing protein	SPSK_03487	7.3	0	15.459
Ornithine aminotransferase	SPSK_01143	5.4	0	15.143
Importin subunit alpha	SPSK_08925	3.8	0	14.942
Parafibromin	SPSK_03365	4.7	0	14.854
DJ-1_PfpI domain-containing protein	SPSK_05530	10	0	14.524
Large subunit ribosomal protein L17e	SPSK_05559	12.7	0	14.458
Phosphotransferase	SPSK_08897	3.5	0	14.44

ATP synthase subunit 4, mitochondrial	SPSK_00863	6	0	14.239
Uncharacterized protein	SPSK_10287	6.4	0	13.952
Plasminogen activator inhibitor 1 RNA-binding protein	SPSK_02483	5.3	0	13.881
Adenylate kinase	ADK1	7.3	0	13.873
Eukaryotic translation initiation factor 5A	SPSK_00554	15.2	0	13.804
ATP synthase subunit delta, mitochondrial	SPSK_02676	9.3	0	13.679
Guanine nucleotide-binding protein subunit beta-2-like 1 protein	SPSK_02542	5.1	0	13.639
Actin-related protein 2/3 complex subunit 5	SPSK_09785	7.2	0	13.619
60S ribosomal protein L36	SPSK_05546	15.4	0	13.603
BAR domain-containing protein	SPSK_01164	5.6	0	13.563
1,4-alpha-glucan-branching enzyme	SPSK_01450	2.4	0	13.542
Acetyl-CoA C-acetyltransferase	SPSK_00811	4.4	0	13.479
Aminoacyl tRNA synthase complex-interacting multifunctional protein 1	SPSK_09365	4.4	0	13.459
Large subunit ribosomal protein L34e	SPSK_07894	6.9	0	13.386
Adenylosuccinate synthetase	SPSK_08779	3.4	0	13.336
Saccharopine dehydrogenase [NAD(+), L-lysine-forming]	SPSK_03427	6.1	0	13.306
RRM domain-containing protein	SPSK_05242	13.6	0	13.288
Large subunit ribosomal protein L23Ae	SPSK_02614	16.6	0	13.268
NADH:ubiquinone oxidoreductase 21.3kD subunit 21.3a	SPSK_06245	10	0	13.158
Altered inheritance of mitochondria protein 41	AIM41	11.4	0	13.14
2-oxoisovalerate dehydrogenase E1 component, beta subunit	SPSK_09224	3.4	0	13.138
Coronin	SPSK_08512	3	0	12.92
Uncharacterized protein	SPSK_10985	3.2	0	12.902
Succinate--CoA ligase [ADP-forming] subunit alpha, mitochondrial	SPSK_07522	6	0	12.888
40S ribosomal protein S21	SPSK_10808	17.2	0	12.875
Histone-glutamine methyltransferase	SPSK_05361	9.1	0	12.872
Obg-like ATPase 1	SPSK_09160	4.8	0	12.847
Mitochondrial processing peptidase	SPSK_05173	3.4	0	12.845
Uncharacterized protein	SPSK_05019	2.3	0	12.829
Glyoxalase II	SPSK_10577	8.6	0	12.766
Uncharacterized protein	SPSK_06078	3.2	0	12.689
3-hydroxyisobutyrate dehydrogenase	SPSK_05342	5.9	0	12.658
Peptidyl-prolyl cis-trans isomerase	SPSK_09644	9.9	0	12.648
Small subunit ribosomal protein S27Ae	SPSK_04603	9.7	0	12.449

Thioredoxin 1	SPSK_05618	8.6	0	12.305
Mannitol-1-phosphate 5-dehydrogenase	SPSK_03429	4	0	12.146
Histone-binding protein RBBP4	SPSK_05168	3.4	0	12.088
SCP2 domain-containing protein	SPSK_05584	12	0	12.058
H(+)-transporting two-sector ATPase	SPSK_04862	1.6	0	11.819
Uncharacterized protein	SPSK_03697	11.1	0	11.782
Uncharacterized protein	SPSK_02866	32.4	0	11.728
Uncharacterized protein	SPSK_04635	13.7	0	11.567
Uncharacterized protein	SPSK_09471	6	0	11.517
Histone H4	SPSK_09880	19.4	0	11.392
Glucose-methanol-choline (Gmc) oxidoreductase	SPSK_06158	3.3	0	11.277
Tubulin beta chain	SPSK_01580	33.3	0	11.237
26S proteasome regulatory subunit RPN11	SPSK_09493	7.1	0	11.101
Glucoamylase	SPSK_10517	5.2	0	11.089
Uncharacterized protein	SPSK_04796	0.4	0	11.086
Conserved lysine-rich protein	SPSK_08828	3.1	0	10.941
Uncharacterized protein	SPSK_03325	4.5	0	10.92
2-oxoglutarate dehydrogenase E1 component	SPSK_01410	1.6	0	10.749
G2 m phase checkpoint control protein	SPSK_02499	4.7	0	10.562
Eukaryotic translation initiation factor 3 subunit B	PRT1	2.4	0	10.551
Isocitrate dehydrogenase [NAD] subunit, mitochondrial	SPSK_01714	3.9	0	10.467
Glycerol 3-phosphatase 1	SPSK_06510	6.2	0	10.203
Large subunit ribosomal protein L13e	SPSK_01270	9.5	0	10.136
Enoyl-CoA hydratase	SPSK_09558	4.1	0	10.05
Uncharacterized protein	SPSK_09263	3	0	9.9252
3-methylcrotonyl-CoA carboxylase alpha subunit	SPSK_08659	2.5	0	9.6968
Alpha-NAC	SPSK_03592	6.4	0	9.5562
Amidase domain-containing protein	SPSK_00743	2.1	0	9.4135
F-type H+-transporting ATPase subunit alpha	SPSK_04513	12.4	0	9.0877
NADH-ubiquinone oxidoreductase	SPSK_02743	7.3	0	9.0858
Rnapii degradation factor	SPSK_06950	1.3	0	9.0779
U6 snRNA-associated Sm-like protein LSm5	LSM5	15	0	8.9445
Cytochrome c oxidase subunit 2	SPSK_11049	3.2	0	8.8964
FACT complex subunit POB3	SPSK_07192	2.6	0	8.896
TPR_REGION domain-containing protein	SPSK_06357	7.7	0	8.8485
MEI5 protein	SPSK_06060	1.9	0	8.8447

Trehalase	SPSK_03113	1.7	0	8.7682
BolA domain protein	SPSK_00627	14.6	0	8.6157
GrpE protein homolog	SPSK_01874	4	0	8.5419
Uncharacterized protein	SPSK_02801	7.1	0	8.506
Uncharacterized protein	SPSK_05105	0.9	0	8.4894
ATP synthase subunit e, mitochondrial	SPSK_00909	12.5	0	8.4779
Signal sequence receptor alpha chain	SPSK_04413	5.8	0	8.4046
Large subunit ribosomal protein L4e	SPSK_05336	3.2	0	8.2417
Acetyl-CoA C-acetyltransferase	SPSK_05258	5.5	0	8.0483
U3 small nucleolar RNA-associated protein 21	SPSK_00389	0	0.003817	8.0299
Nucleolar protein 58	SPSK_04074	2.2	0.003802	8.0294
Small subunit ribosomal protein S2e	SPSK_03495	4.2	0.003788	7.9761
Histone-lysine N-methyltransferase, H3 lysine-79 specific	SPSK_07317	2.9	0.003774	7.9714
Ran GTPase-activating protein 1	SPSK_09315	3.3	0.003759	7.9629
Uncharacterized protein	SPSK_05527	6	0.003745	7.9458
Cytochrome c domain-containing protein	SPSK_01853	10.9	0.003731	7.8203
Isocitrate dehydrogenase [NADP]	SPSK_09014	2.6	0.003718	7.8146
40S ribosomal protein S6	SPSK_09576	3.4	0.003704	7.7156
Actin-related protein 2/3 complex subunit 3	SPSK_09786	5.6	0.00369	7.6797
40S ribosomal protein S25	SPSK_01265	14.6	0.003677	7.6761
T-complex protein 1 subunit epsilon	SPSK_05111	5.4	0.003663	7.6282
1,3-beta-glucanosyltransferase	SPSK_05276	3.1	0.00365	7.6249
NADH dehydrogenase (Ubiquinone) 1 alpha subcomplex 9	SPSK_00506	3.7	0.003636	7.4729
Pyruvate decarboxylase	SPSK_02280	1.5	0.003623	7.4286
U6 snRNA-associated Sm-like protein LSm3	LSM3	10.9	0.00361	7.4075
3-methylcrotonyl-CoA carboxylase beta subunit	SPSK_08853	3.3	0.003597	7.4007
DNA topoisomerase 2-associated protein PAT1	SPSK_08536	1.3	0.003584	7.3431
60S acidic ribosomal protein P0	SPSK_04464	3.8	0.003571	7.3012
Arginase	SPSK_04404	3	0.003559	7.2623
Covalently-linked cell wall protein	SPSK_01786	3	0.003546	7.2527
Uncharacterized protein	SPSK_10180	12.5	0.003534	7.2322
Sec14 cytosolic factor	SPSK_00537	4.3	0.003521	7.1405
Large subunit ribosomal protein L23e	SPSK_07984	4.6	0.007018	7.1137
F-type H+-transporting ATPase subunit h	SPSK_10019	9.3	0.006993	7.0513
Iron sulfur cluster assembly protein mitochondrial	SPSK_10709	7.9	0.006969	7.0233
4-aminobutyrate aminotransferase	SPSK_05199	2.1	0.006944	7.0132

Uncharacterized protein	SPSK_06752	17.8	0.00692	6.9967
Transcription initiation factor IIF subunit beta	SPSK_00815	3.1	0.006897	6.9427
Kh domain protein RNA-binding protein	SPSK_02555	3.2	0.006873	6.8979
Uncharacterized protein	SPSK_08634	1.3	0.006849	6.8949
Uncharacterized protein	SPSK_07466	3.1	0.006826	6.7471
Uncharacterized protein	SPSK_09019	1.9	0.006803	6.6787
T-complex protein 1 subunit zeta	SPSK_07242	1.7	0.00678	6.6521
Elongation factor Tu	SPSK_05297	2	0.006757	6.6454
Uncharacterized protein	SPSK_01583	2.3	0.006734	6.6297
Protein disulfide-isomerase A6	SPSK_05269	2.7	0.006711	6.6281
Helicase C-terminal domain-containing protein	SPSK_08039	8.3	0.006689	6.6273
ATP-dependent Clp protease ATP-binding protein subunit ClpB	SPSK_08586	1.2	0.006667	6.6258
Glutamate decarboxylase	SPSK_04159	1.9	0.006645	6.6123
Omp85 domain-containing protein	SPSK_07325	1.8	0.006623	6.6123
Centromere-binding protein	SPSK_05220	2.1	0.006601	6.6053
Dihydrolipoamide acetyltransferase component of pyruvate dehydrogenase complex	SPSK_09017	2.3	0.006579	6.5833
Probable endonuclease LCL3	SPSK_08520	1.5	0.006557	6.581
5-aminoimidazole-4-carboxamide ribonucleotide formyltransferase	SPSK_08790	1.9	0.006536	6.5558
Prolyl-tRNA synthetase	SPSK_08956	3.8	0.006515	6.5283
Tyrosine 3-monooxygenase/tryptophan 5-monooxygenase activation protein	SPSK_05465	12.2	0.006494	6.5156
Suppressor of G2 allele of SKP1	SPSK_06242	2.2	0.006473	6.5023
Uncharacterized protein	SPSK_04914	1.2	0.006452	6.5023
Catalase	SPSK_07775	2.8	0.006431	6.4568
ATPase GET3	GET3	2.5	0.00641	6.4144
Lysophospholipid acyltransferase	SPSK_02327	0	0.009615	6.4135
3-hydroxyisobutyryl-CoA hydrolase	SPSK_04045	1.8	0.009585	6.4063
Glutathione S-transferase	SPSK_06547	3.3	0.009554	6.3998
Cys-Gly metallodipeptidase DUG1	SPSK_08962	2.1	0.009524	6.3728
GATA-type domain-containing protein	SPSK_07441	2	0.009494	6.3688
Protoporphyrinogen oxidase	SPSK_08568	1.5	0.009464	6.3679
Cytochrome b-c1 complex subunit 7	SPSK_05690	16.1	0.009434	6.3636
RNA cytidine acetyltransferase	NAT10	1	0.009404	6.3559
Lysine--tRNA ligase	SPSK_01032	1.4	0.009375	6.3514
Eukaryotic translation initiation factor 3 subunit D	SPSK_00750	1.9	0.009317	6.3462
GARP complex component (Vps54)	SPSK_04830	0.8	0.009288	6.3356
Uncharacterized protein	SPSK_07792	6.1	0.009259	6.3047

Mitochondrial import inner membrane translocase subunit TIM10	SPSK_01277	9.6	0.009231	6.2951
Peptide-methionine (S)-S-oxide reductase	SPSK_07987	4.2	0.009203	6.2951
Uncharacterized protein	SPSK_05905	0.6	0.009174	6.2875
Adenosine kinase	SPSK_06388	3.1	0.009146	6.2474
60S ribosomal protein L29	HMPREF1624_01952	12.3	0.009119	6.2462
